# Triggering anti-GBM immune response with EGFR-mediated photoimmunotherapy

**DOI:** 10.1186/s12916-021-02213-z

**Published:** 2022-01-21

**Authors:** Justyna Mączyńska, Florian Raes, Chiara Da Pieve, Stephen Turnock, Jessica K. R. Boult, Julia Hoebart, Marcin Niedbala, Simon P. Robinson, Kevin J. Harrington, Wojciech Kaspera, Gabriela Kramer-Marek

**Affiliations:** 1 Division of Radiotherapy and Imaging, The Institute of Cancer Research, 123 Old Brompton Road, London, SW7 3RP UK; 2 Department of Neurosurgery, Medical University of Silesia, Regional Hospital, 41-200 Sosnowiec, Poland

**Keywords:** Photoimmunotherapy, Glioblastoma, Affibody molecules, IR700

## Abstract

**Background:**

Surgical resection followed by chemo-radiation postpones glioblastoma (GBM) progression and extends patient survival, but these tumours eventually recur. Multimodal treatment plans combining intraoperative techniques that maximise tumour excision with therapies aiming to remodel the immunologically cold GBM microenvironment could improve patients’ outcomes. Herein, we report that targeted photoimmunotherapy (PIT) not only helps to define tumour location and margins but additionally promotes activation of anti-GBM T cell response.

**Methods:**

EGFR-specific affibody molecule (Z_EGFR:03115_) was conjugated to IR700. The response to Z_EGFR:03115_-IR700-PIT was investigated in vitro and in vivo in GBM cell lines and xenograft model. To determine the tumour-specific immune response post-PIT, a syngeneic GBM model was used.

**Results:**

In vitro findings confirmed the ability of Z_EGFR:03115_-IR700 to produce reactive oxygen species upon light irradiation. Z_EGFR:03115_-IR700-PIT promoted immunogenic cell death that triggered the release of damage-associated molecular patterns (DAMPs) (calreticulin, ATP, HSP70/90, and HMGB1) into the medium, leading to dendritic cell maturation. In vivo, therapeutic response to light-activated conjugate was observed in brain tumours as early as 1 h post-irradiation. Staining of the brain sections showed reduced cell proliferation, tumour necrosis, and microhaemorrhage within PIT-treated tumours that corroborated MRI T_2_*w acquisitions. Additionally, enhanced immunological response post-PIT resulted in the attraction and activation of T cells in mice bearing murine GBM brain tumours.

**Conclusions:**

Our data underline the potential of Z_EGFR:03115_-IR700 to accurately visualise EGFR-positive brain tumours and to destroy tumour cells post-conjugate irradiation turning an immunosuppressive tumour environment into an immune-vulnerable one.

**Supplementary Information:**

The online version contains supplementary material available at 10.1186/s12916-021-02213-z.

## Background

Glioblastoma (GBM) is the most common primary malignant brain tumour in adults and is associated with an extremely aggressive clinical course and poor prognosis [1]. The median progression-free survival in primary GBM is 6.9 months, and the median overall survival is 14.6 months with standard-of-care surgery, radiation therapy, and temozolomide [2, 3]. Consequently, there is a high unmet clinical need for new treatment paradigms yielding more durable remissions.

The current neurosurgical management of GBM aims for maximal resection while avoiding additional neurological damage. Numerous methods have been developed to facilitate surgery, including 5-aminolevulinic acid (5-ALA) fluorescence-guided surgery, intraoperative neuro-navigation, and neurophysiological monitoring [4, 5]. However, GBM recurrence is almost inevitable due to residual areas of diffuse microscopic infiltration of tumour cells into the surrounding brain parenchyma and intratumoural heterogeneity at the cellular and molecular levels.

Approximately 57% of GBMs contain a mutation, rearrangement, splicing alteration, and/or amplification of the epidermal growth factor receptor (EGFR). The most common EGFR variant is a deletion of exons 2–7, EGFRvIII, which often co-occurs with focal EGFR amplification, which together are associated with a more aggressive, immuno-evasive tumour phenotype and worse prognosis [6]. Despite the well-known role of EGFR in GBM, the potential of targeting the receptor with tyrosine kinase inhibitors (TKIs) as well as monoclonal antibodies (mAbs) have been unfulfilled so far. Furthermore, a phase III study (ACT IV), for newly diagnosed patients with GBM treated with Rindopepimut, an EGFRvIII-targeted vaccine, also failed to demonstrate a survival benefit [7].

 Interestingly, recent studies have shown that inhibiting EGFR signalling may reduce tumour cell-intrinsic EGFR-induced programmed death-ligand 1 (PD-L1) upregulation, as well as extrinsic IFNγ-induced signals associated with CD8+ T cell infiltration into the tumour microenvironment (TME) [1, 8]. However, attempts to incorporate immune checkpoint inhibitors (ICPIs) into GBM treatment regimens have demonstrated only modest and unpredictable responses [9, 10]. This is most likely due to low burdens of somatic mutations and a relatively immune-depleted (“cold”) GBM microenvironment characterised by a high level of immunosuppressive cytokines (e.g. TGFβ, IL-10) which inhibit immune effector cell activity [11]. Excitingly, several research groups have reported that high-level infiltration of immune effector cell populations, including CD8+ cytotoxic T-lymphocytes (CTLs), into the TME can improve response to ICPIs in GBM [12, 13]. Therefore, in a clinical context, it would be desirable to restore intratumoural infiltration of CD8+ T cells to create an immunologically “hot” TME and, thus, promote the responsiveness of GBM to ICPIs.

One way to activate the TME immunologically would be through the use of photoimmunotherapy (PIT) and conventional photodynamic therapy (PDT).

PIT is a light-mediated therapeutic approach, where a photosensitiser (PS) is conjugated to a highly specific monoclonal antibody (mAb), antibody fragment, or affibody molecule that has the ability to engage the selected target of interest. Near-infrared (NIR) light irradiation of the conjugate lead to ligand release reaction of IR700 and under normoxic conditions to the production of heat and reactive oxygen species (ROS) that, consequently, initiate target-selective cell death and stimulate inflammation, followed by vascular shutdown and tissue ischaemia [14–16]. For example, Nagaya et al. have shown that anti-CD44-IR700-mediated PIT can significantly delay tumour growth following a single treatment in three CD44-expressing syngeneic mouse models of oral squamous cell carcinoma [17]. In addition, NIR-PIT targeting EGFR with anti-can225-IR700 resulted in rapid cell death in vitro and tumour growth inhibition in vivo, improving mouse survival [18]. More importantly, EGFR-targeting IR700-cetuximab (ASP-1929, Akalux™, Rakuten Medical, Inc.) is currently being investigated in a global phase III clinical trial in head and neck cancer (NCT03769506) [19] and was registered for clinical use in Japan [20].  Furthermore, it has been shown that both PIT and PDT can trigger immunogenic cell death (ICD), as exemplified by the release of damage-associated molecular patterns (DAMPs), including calreticulin (CRT), heat shock proteins HSP70/90, ATP, and high-mobility group box-1 (HMGB1) nuclear protein that subsequently activate immune cells upon binding to pattern recognition receptors [21].

In view of the high expression rate and oncogenic nature of EGFR, we have postulated that PIT targeting this receptor could promote CD8+ T cell attraction and activation and overcome the immunologically “cold” status of GBM.

As an alternative to full-size antibodies, we have previously investigated the smaller, IR700-labelled EGFR-specific affibody molecule (Z_EGFR:03115_-IR700), aiming for more effective tumour penetration, faster delivery, and clearance from non-targeted tissues [22]. After demonstrating that Z_EGFR:03115_-IR700 cell uptake enables imaging of EGFR expression in an orthotopic brain tumour model (U87-MGvIII), our proof-of-concept in vivo PIT study also showed the conjugate’s therapeutic efficacy in subcutaneous glioma xenografts [22].

In the current study, we report that Z_EGFR:03115_-IR700-PIT promotes the production of DAMPs from cancer cells, also leading to dendritic cell (DC) maturation in vitro. In addition, when applied in a syngeneic mouse model, the treatment induces T cell responses that might overcome the “immunologically cold” status of GBM. Therefore, we believe that this therapeutic approach, following complete or cytoreductive resection of GBM, could lead to (i) elimination of residual or surgically inaccessible EGFR+ve cancer cells and (ii) subsequent stimulation of anti-tumour immunity.

## Methods

### Preparation of Z_EGFR:03115_-IR700

The conjugation of IRDye700DX-maleimide (IR700, ex. 689 nm, em. 700 nm; LI-COR® Bioscience, USA) to the Z_EGFR:03115_-Cys affibody molecules (Affibody, Sweden) is described in detail in the supporting information (Additional File 1).

### Cell lines and cell culture

Human GBM cell line DKMG and murine GBM cell line GL261 were purchased from the Celther Polska (Poland) and the German Collection of Microorganisms and Cell Cultures (DSMZ, Germany), respectively. U87-MG and U87-MGvIII were kindly provided by Dr. Frank Furnari (Ludwig Cancer Research, USA) [23]. The primary, patient-derived cell lines WSz4, WSz50, and WSz57 have been recently established in our lab [22]. The cells were grown as described in the supporting information. BL6-NPE-GFP-Luc murine GBM cell line was kindly provided by Dr. Steven Pollard (University of Edinburgh, UK) and cultured as previously reported [24]. The genetic origin of all the cell lines was tested and authenticated by short tandem repeat (STR) DNA profiling analysis (Eurofins Medigenomix, Germany). The cells were also routinely tested and found to be negative for *Mycoplasma* contamination (PCR detection kit, Surrey Diagnostics Ltd., UK).

### Singlet oxygen production assay

Singlet oxygen (^1^O_2_) production was determined using the Singlet Oxygen Sensor Green reagent (SOSG, Thermo Fisher Scientific, UK) according to the protocol provided by the manufacturer. More details about the assay are described in the supporting information.

### Cellular binding of Z_EGFR:03115_-IR700

Human and murine GBM cells were harvested and incubated in a medium with Z_EGFR:03115_-IR700 (30 nM) for 1 h at 4 °C, and samples were analysed using flow cytometry (BD™ LSRII). To test the targeting specificity and internalisation of the conjugate, cells were plated on confocal glass-bottomed dishes (Thermo Fisher Scientific, USA) in complete medium with Z_EGFR:03115_-IR700 (1 μM) for 1 h at 4 °C or 1, 3, and 6 h at 37 °C and analysed using a Zeiss LSM700 confocal microscope (Carl Zeiss Inc., Germany). A detailed description of the procedures is given in the supporting information.

### In vitro PIT studies

U87-MGvIII cells were seeded on petri dishes 24 h before experiments. Afterwards, cells were incubated with Z_EGFR:03115_-IR700 (0.1 to 1 μM) for 1, 3, or 6 h at 37 °C. The media were then changed for phenol red-free DMEM medium and cells irradiated (8 or 16 J/cm^2^) using a LED light source (L690−66−60, Marubeni America Co., USA). Cell viability was determined using the CellTiter-Glo® (Promega, USA) luminescent assay 24 h post-light exposure. To assess ROS production, 5 μM 2′,7′-dichlorofluorescein diacetate (DCFDA; Sigma, UK) was added to phenol-red free medium during irradiation. The cell death at 1, 4, and 24 h post-irradiation was assessed using the Annexin V/Dead Cell Apoptosis Kit (Thermo Fisher Scientific, UK) according to the manufacturer’s instruction. To determine the post-PIT ATP and HMGB1 release, the ENLITEN® ATP assay (Promega, USA) and an HMGB1 ELISA kit (Tecan, IBL International, Germany) were used. Calreticulin exposure on the membrane was measured by flow cytometry (BD™ LSRII). All the methods are described in detail in the supporting information.

### Co-culture with dendritic cells

The experimental details about co-culturing the immature dendritic cells (iDCs) with PIT-treated U87-MGvIII or DKMG cells are given in the supporting information.

### Western blot

Western blotting was performed as previously described [22]. Proteins released into the medium were extracted using an acetone precipitation protocol (Thermo Fisher Scientific, USA). The list of antibodies used and densitometric analysis are provided in the supporting information.

### ^18^F-AlF-NOTA-Z_EGFR:03115_ preparation

The preparation of NOTA-Z_EGFR:03115_ and its radiolabelling with the ^18^F-Al complex was performed as previously described [25].

### In vivo studies

All experiments were performed in compliance with licences issued under the UK Animals (Scientific Procedures) Act of 1986 and following local ethical review. Studies were compliant with the UK National Cancer Research Institute Guidelines for Animal Welfare in Cancer Research [26] and the ARRIVE (animal research: reporting in vivo experiments) guidelines [27].

### Mouse models

The detailed methods are described in the supporting information. Briefly, NCr athymic female mice (5–6 weeks) were bred in-house. C57BL/6J female mice (6–7 weeks) used for the syngeneic model, were purchased from Charles River, UK. The orthotopic GBM U87-MGvIII or BL6-NPE-GFP-Luc mouse models were established as previously described [22, 24]. For the subcutaneous GBM xenografts, U87-MGvIII cells were injected over the right shoulder. Once tumours reached approximately 60 mm^3^, mice were randomly distributed into the experimental groups.

### PIT in vivo

For PIT treatment studies, subcutaneous and intracranial GBM U87-MGvIII xenografts were randomised into the following treatment groups: (i) light exposure only (100 J/cm^2^) and (ii) 18 μg Z_EGFR:03115_-IR700 with light exposure (100 J/cm^2^, 0.0886 W/cm^2^). For immunocompetent mice bearing intracranial tumours, 50 J/cm^2^ light dose was used. The tumours were irradiated with a LED light source (L690−66−60, peak 690 ± 20 nm) 1 h post-conjugate i.v. injection. More details are provided in the supporting information.

### MR imaging

To monitor the orthotopic tumour growth, mice were imaged using the 1 T M3™ MRI system (Aspect Imaging, Israel) with a T_2_-weighted imaging sequence and a dedicated head coil. To perform high-resolution acquisitions, mice were scanned using the 7 T Biospec® horizontal micro-imaging system (Biospec®, Bruker, Germany). The imaging protocols are described in the supporting information.

### PET imaging

Mice (*n* = 5) with MRI-confirmed brain tumours received an i.v. injection of ^18^F-AlF-NOTA-Z_EGFR:03115_ (12 μg; 2.4 ± 0.15 MBq/mouse), and PET/CT scans were acquired 1, 3, and 5 h post-injection of the radiotracer using an Albira PET/SPECT/CT imaging system. The detailed imaging and data analysis protocols are given in the supporting information.

### Autoradiography

Dissected tumour and brain tissue samples were collected and immediately embedded in an optimal cutting temperature compound (Tissue-Tek® O.C.T, Netherlands) and snap-frozen in liquid nitrogen.

Further experimental details are given in the supporting information.

### Fluorescent imaging

In vivo and ex vivo fluorescence images were acquired as stated in the supporting information using an IVIS Spectrum/CT system (Perkin Elmer, USA).

### Immunohistochemistry

Formalin-fixed brain and tumour tissues were embedded in paraffin, sectioned (5-μm-thick slices), and mounted on microscope slides. Frozen embedded tissues were sectioned into 10-μm-thick slices and mounted on microscope slides before being fixed in ice-cold acetone. The detailed staining procedures with the various antibodies are described in the supporting information.

### Tumour and T cell isolation

Tumour and surrounding brain tissue were harvested and dissociated via enzymatic digestion (Liberase TL, Roche, Switzerland). Single-cell suspension was prepared by straining the digested tissue through a 70-μm mesh. Further experimental details are given in the supporting information.

### Serum cytokine analysis

The serum was separated from the whole blood collected from the mice at the 24 h endpoint, snap-frozen, and stored at − 80 °C until further analysis. Concentrations of various cytokines were analysed using a Mouse Cytokine Proinflammatory Focused 10-plex Array (Eve Technologies, Canada).

### Statistical analysis

Unless otherwise stated, data were expressed as the mean ± SD. Statistical significance, sample size calculations, and correlation analysis are described in detail in the supporting information.

## Results

### Z_EGFR:03115_-IR700-PIT leads to an EGFR expression-dependent response in vitro

**Fig. 1:**
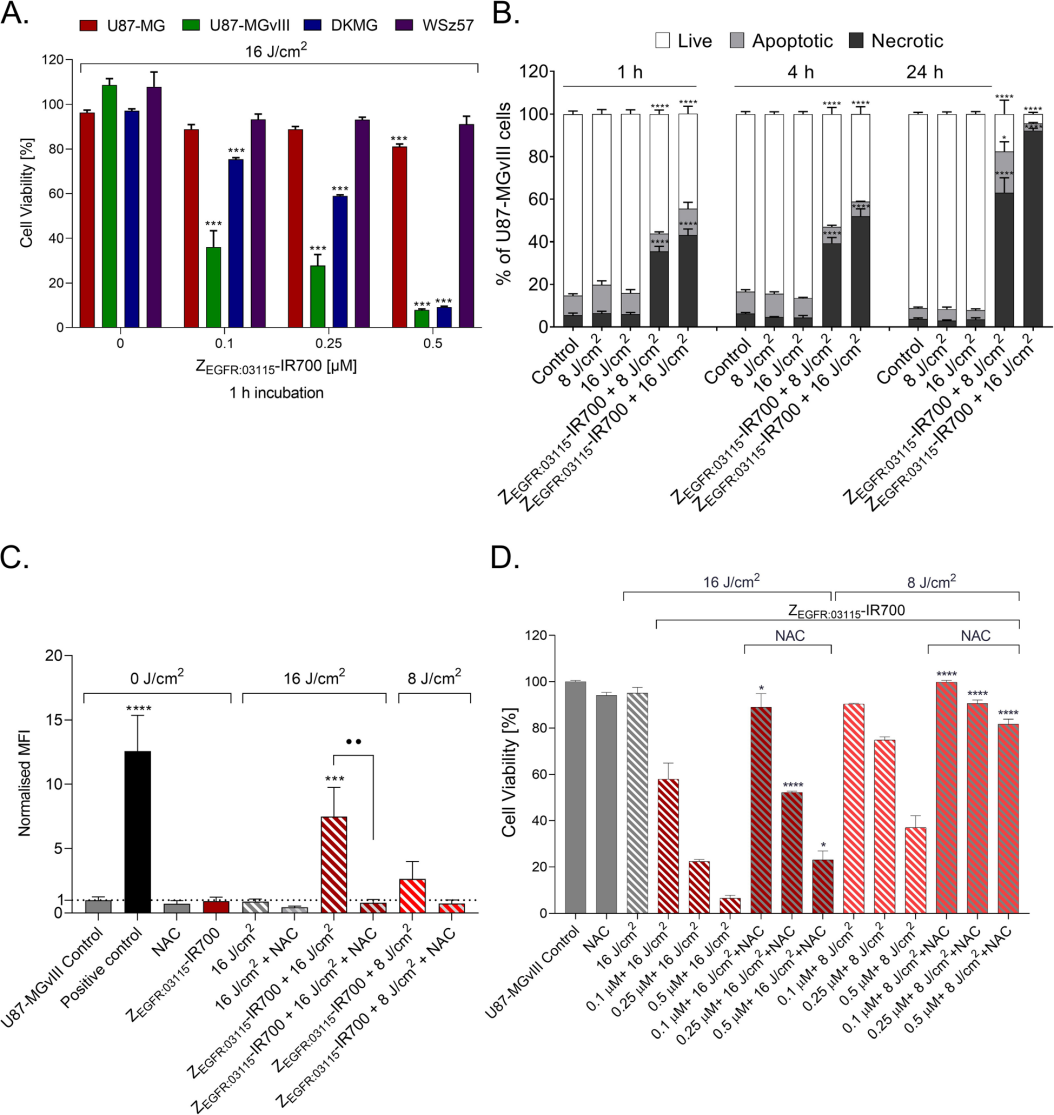
Z_EGFR:03115_-IR700-based PIT in vitro. **A** Decrease in cell viability as assessed by the CellTiter-Glo® viability assay 24 h post-treatment in GBM cells (U87-MG, U87-MGvIII DKMG, and WSz57) following 1 h incubation with the Z_EGFR:03115_-IR700 (0.1–0.5 μM) and irradiation with 16 J/cm^2^ light dose compared to control cells. The results were normalised to the untreated cells (no light, no conjugate) and presented as mean ± SEM (*n* = 3). Statistical difference in comparison with the control determined using ANOVA with Dunnett’s post hoc test. ****p ≤ 0.0001, ***p ≤ 0.001. **B** Changes in the percentage of live, apoptotic, and necrotic U87-MGvIII cell populations measured 1, 4, or 24 h post-therapy, following 1 h incubation with Z_EGFR:03115_-IR700 (0.25 μM) and exposure to 8 or 16 J/cm^2^ light dose. Data are presented as mean ± SEM (*n* = 3). Statistical significance in comparison with the control (untreated) was determined using ANOVA with Dunnett’s post hoc test. ****p ≤ 0.0001, ***p ≤ 0.001, *p ≤ 0.05. **C** Reactive oxygen species (ROS; using DCFDA) production in U87-MGvIII cells, evaluated 10 min post-treatment: 1 h incubation with or without Z_EGFR:03115_-IR700 (0.25 μM), *N*-acetyl-l-cysteine (NAC, 5 mM) and irradiation (8 or 16 J/cm^2^) compared to control cells; 50 μM TBHP was used as a positive control. The results were normalised to the control and presented as mean ± SEM (*n* = 3). Statistical difference in comparison with the control determined using ANOVA with Dunnett’s post hoc test. ****p ≤ 0.0001, ***p ≤ 0.001. ^••^*p* ≤ 0.01 significant decrease in ROS generation after NAC incubation of PIT treated cells. **D** Decrease in cell viability as assessed by the CellTiter-Glo® viability assay 24 h post-treatment in U87-MGvIII cells, following 1 h incubation with Z_EGFR:03115_-IR700 (0.1–0.5 μM), with or without *N*-acetyl-l-cysteine (NAC, 5 mM) and irradiation with 8 or 16 J/cm^2^ light dose, was confirmed to be dose-dependent and ROS-mediated. Data are presented as mean ± SEM (*n* = 3). Statistical difference between the groups with or without NAC incubation was determined using the Mann-Whitney test. ****p ≤ 0.0001, *p < 0.05

The affibody molecule (Z_EGFR:03115_), which recognises the murine and human extracellular epitope of EGFR, was conjugated to IR700. Specific and receptor expression-dependent Z_EGFR:03115_-IR700 binding (Fig. S1A), as measured by flow cytometry, was in line with the total EGFR level assessed via Western blot (Fig. S1B) in a panel of human and mouse GBM cell lines. In order to confirm that Z_EGFR:03115_-IR700 PIT induces target-specific cell death, U87-MGvIII (EGFR high), DKMG (EGFR high), WSz57 (EGFR medium), and U87-MG (EGFR low) cells were incubated with increasing concentrations of the conjugate (0–0.5 μM; 1 h) and exposed to dose of NIR light selected based on our previous studies [22]. A significant decrease in cell viability in a conjugate concentration-dependent manner was seen in both U87-MGvIII and DKMG cells 24 h post-irradiation with 16 J/cm^2^ (Fig. 1A). However, DKMG appeared to be more resistant to the treatment in the presence of low concentrations of the conjugate (survival 75% and 59% at 0.1 and 0.25 μM, respectively) compared to U87-MGvIII cells (survival 36% and 28%, respectively). At the highest concentration of Z_EGFR:03115_-IR700 (0.5 μM), both cell lines demonstrated a dramatic loss in cell viability (less than 10% survival). A small but significant reduction in cell viability (81% survival) was observed in U87-MG cells when the highest concentration of the conjugate was tested (0.5 μM). The patient-derived WSz57 cell line was less sensitive to the treatment, most likely due to highly heterogeneous EGFR expression (Fig. S1C). A longer incubation (6 h) of U87-MG, DKMG, and WSz57 cells with the Z_EGFR:03115_-IR700 (Fig. S1D) led to an enhanced PIT-mediated cell death that was in line with an increased internalisation of the conjugate as confirmed via confocal microscopy (Fig. S1E).

Further, to determine the mechanism of cell death following the treatment, we used the Annexin V/PI assay. Flow cytometric analysis showed that U87-MGvIII cells were dying rapidly, and within 1 h post-irradiation (0.25 μM conjugate, 8 or 16 J/cm^2^), there were three distinct cell populations: viable (Annexin^−^/PI^−^; 45-56%), apoptotic (Annexin^+^/PI^−^; 8–12%), and necrotic (Annexin^+^/PI^+^; 35–43%). Importantly, by 24 h post-irradiation, the population of viable cells was 17.5% and 4% (Z_EGFR:03115_-IR700 + 8 J/cm^2^ or 16 J/cm^2^ delivered light, respectively) compared to the control groups (91%) (Fig. 1B).

It is well recognised that an essential component of the intracellular pathways that enables ICD and the release of DAMPs is ROS production [21]. Therefore, we subsequently investigated whether Z_EGFR:03115_-IR700 PIT-mediated generation of ROS will trigger the activation and trafficking of DAMPs to the extracellular space in vitro. The capability of Z_EGFR:03115_-IR700 to induce singlet oxygen (^1^O_2_) after NIR light activation was initially measured in cell-free conditions. The studies confirmed a significant light dose-dependent SOSG fluorescence enhancement post-irradiation (Fig. S1F). Moreover, U87-MGvIII cells subjected to Z_EGFR:03115_-IR700-based PIT (0.25 μM; 16 J/cm^2^) showed a prominent increase of intracellular ROS production that was significantly suppressed by the ROS scavenger NAC (Fig. 1C). This quenching effect consequently resulted in an inhibition of PIT-induced cell death (Fig. 1D). After PIT with just 8 J/cm^2^, only a slight enhancement in ROS generation was measured in U87-MGvIII cells (Fig. 1C). However, NAC successfully inhibited PIT-induced cell death under each conjugate concentration (Fig. 1D).

### PIT induces the production of DAMPs in GBM cell lines and maturation of iDCs in vitro

**Fig. 2:**
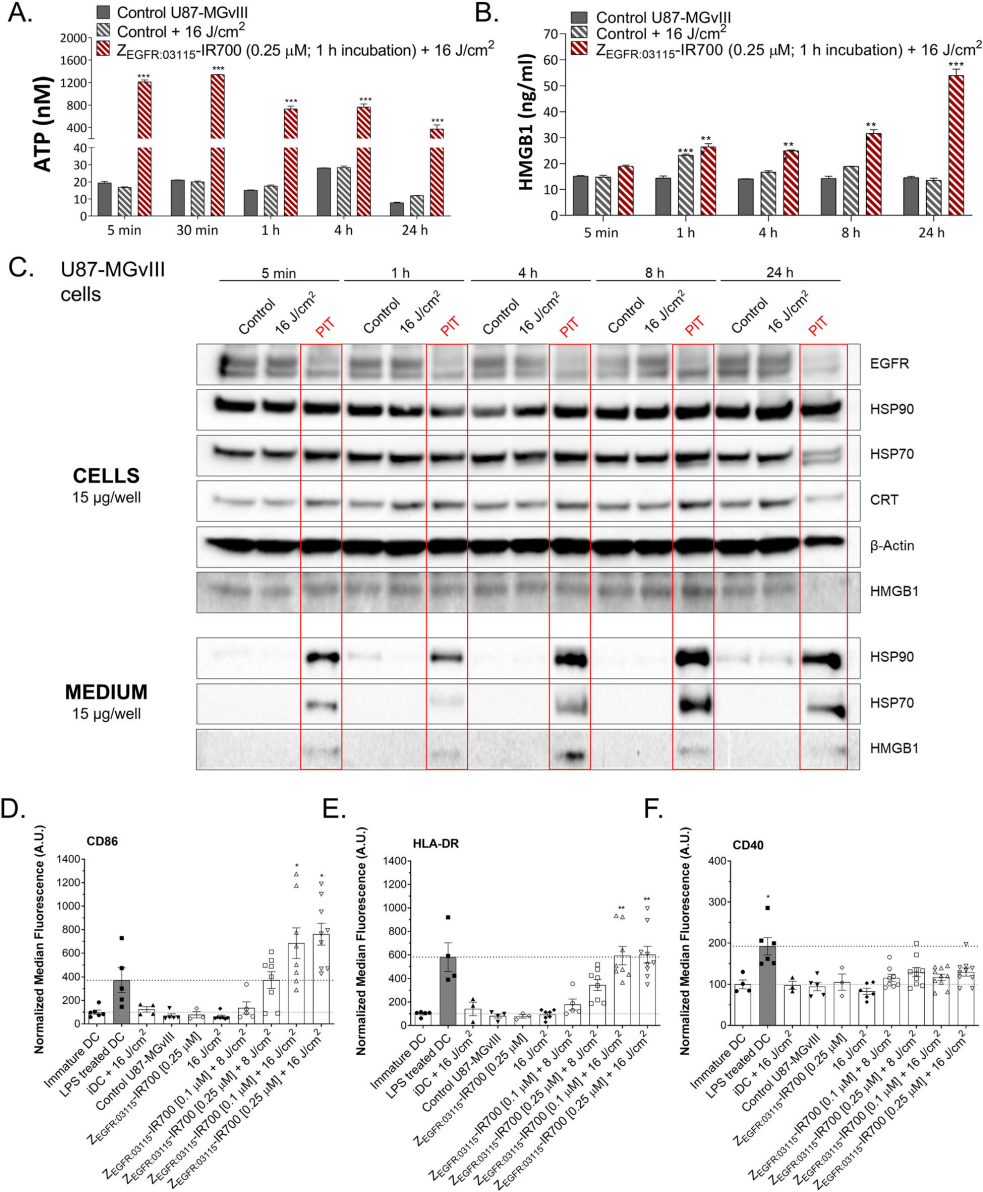
Immunogenic cell death (ICD) of GBM cells following EGFR-targeted PIT. **A**, **B** Concentrations of ATP and HMGB1 proteins released into the medium from U87-MGvIII cells over time (5, 30 min; 1, 4, or 24 h) post-treatment (1 h incubation with or without Z_EGFR:03115_-IR700 (0.25 μM) and irradiation (16 J/cm^2^)). Data are presented as mean ± SEM (*n* = 3). Statistical significance in comparison with the control (untreated) group was determined using ANOVA with Dunnett’s post hoc test. ***p ≤ 0.001. **C** Western blot assessment of EGFR, HSP70, HSP90, calreticulin (CRT) and HMGB1 expression levels in U87-MGvIII cells and cell supernatants (medium) over time (5 min; 1, 4, 8, or 24 h) post-treatment (PIT: 0.25 μM Z_EGFR:03115_-IR700 + 16 J/cm^2^) in comparison with irradiated only (16 J/cm^2^) and control cells. β-Actin was used as a loading control. **D**, **F** DC maturation after co-culturing with Z_EGFR:03115_-IR700-treated (1 h incubation, 0.25 μM) U87-MGvIII cells post-irradiation with 16 J/cm^2^ light dose. CD86, HLA-DR, and CD40 expression level on the surface of DC cell membrane (live, CD14-negative and CD11c-positive population) as measured by flow cytometry 48 h post-treatment. Immature DCs (iDC) cultured without stimulation for maturation were used as a control. *E. coli* lipopolysaccharide (LPS)-treated iDC were used as a positive control. Data are presented as mean ± SEM (*n* = 3–4). The graphs represent the data from four healthy blood donors. Statistical significance in comparison with the iDC group was determined using ANOVA with the Holm-Sidak correction test. **p ≤ 0.01, *p ≤ 0.05

Next, we measured the efficacy of Z_EGFR:03115_-IR700-based PIT (0.25 μM; 16 J/cm^2^) to lead to DAMPs (CRT, HMGB1, HSP70, and HSP90) release in U87-MGvIII cells. As shown in Fig. S2A, there was a transient but significant increase in CRT expression level as early as 5 min post-NIR irradiation. We also observed a rapid (5 min) secretion of ATP into the cell culture media post-irradiation (Fig. 2A). Western blot densitometric analysis (Fig. S2) revealed a rapid release of HMGB1, HSP90, and HSP70 into the culture medium after PIT. These strong immunogenic signals were in line with the pronounced U87-MGvIII PIT-induced cell death (Fig. 1A, 0.25 μM; 16 J/cm^2^) and most likely high sensitivity of these cells to oxidative stress. No other DAMP upregulation was detected in any of the control groups (Fig. 2B; Fig. S2). Additionally, ELISA results (corroborated by Western blot data) showed that cells irradiated 1 h after Z_EGFR:03115_-IR700 treatment released HMGB1 in a time-dependent manner when compared to the control cells (Fig. 2C). We then investigated whether the enhanced levels of ICD markers, induced by Z_EGFR:03115_-IR700 PIT in U87-MGvIII cells, would trigger phenotypic maturation of DCs in a similar manner to conjugates reported by others [28]. We co-cultured Z_EGFR:03115_-IR700-PIT-treated U87-MGvIII cells with iDCs for 48 h. Subsequent flow cytometry analysis showed a significant increase in the expression of CD86 (Fig. 2D) and MHC class II (HLA-DR) (Fig. 2E) molecules on the surface of DCs exposed to PIT-treated U87-MGvIII cells compared to controls. However, there was no change in the CD40 expression level (Fig. 2F).

### Imaging EGFR expression in orthotopic GBM tumours

**Fig. 3:**
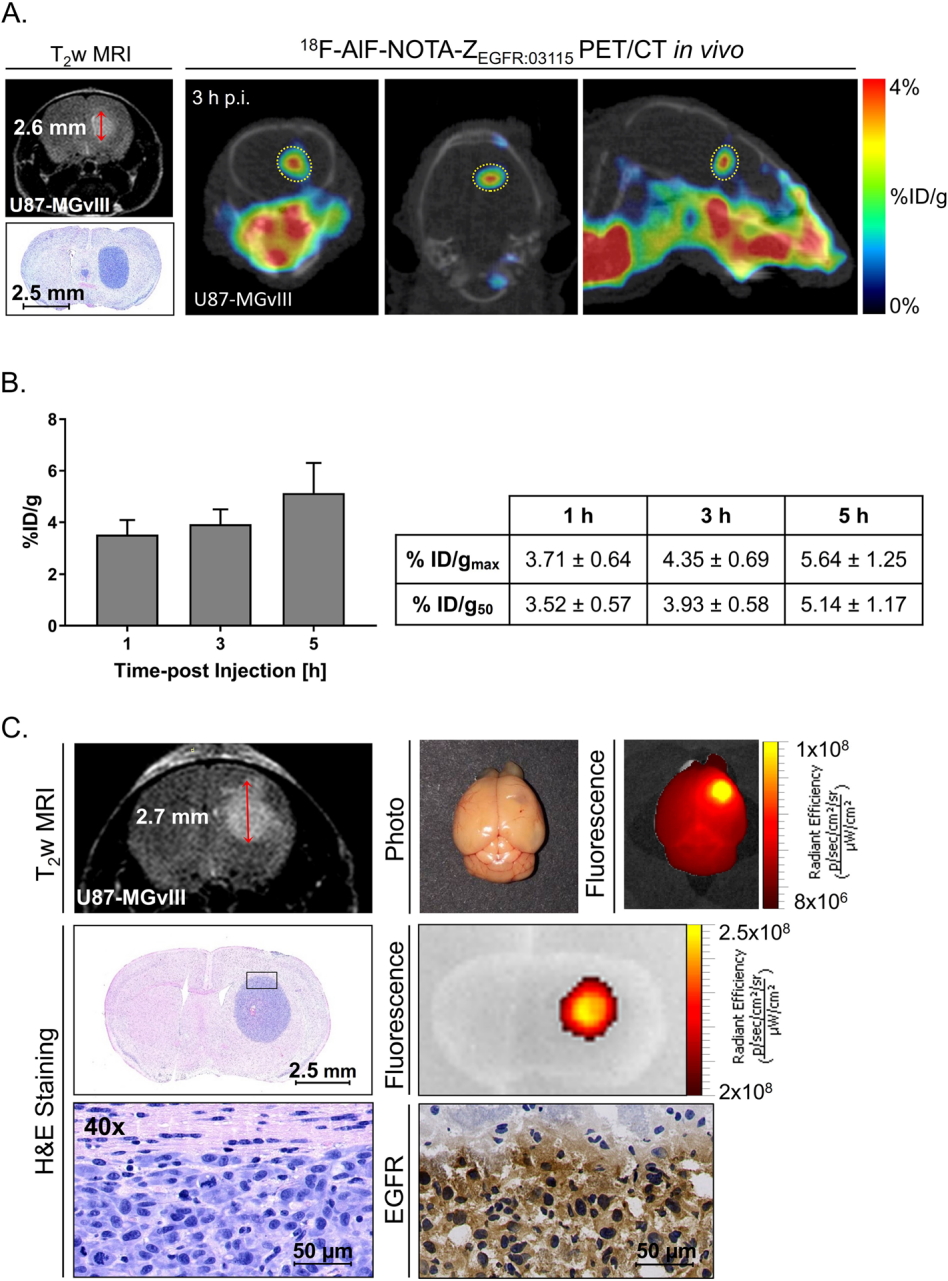
Characterisation of the orthotopic U87-MGvIII model. **A** In vivo axial T_2_-weighted MRI image and corresponding axial, coronal, and sagittal PET/CT images of the orthotopic U87-MGvIII tumour 3 h post-injection of the ^18^F-AlF-NOTA-Z_EGFR:03115_ compared to the haematoxylin/eosin staining. **B** In vivo uptake values 1, 3, and 5 h after i.v. injection of the radiotracer (mean ± SEM), measured as % ID/g_50_ and % ID/g_max_. **C** In vivo axial T_2_-weighted MRI image and corresponding ex vivo photography and fluorescence image 5 days after tumour cell engraftment (tumour diameter, 2.7 mm). Brain collection and fluorescence imaging were performed 1 h after i.v. injection of 18 μg of Z_EGFR:03115_-IR700. Haematoxylin/eosin staining and near-infrared image of Z_EGFR:03115_-IR700 were performed on the consecutive brain sections. The EGFR immunostaining confirmed the high level of EGFR

The capability of Z_EGFR:03115_ to target EGFR-expressing cells in vivo was evaluated using an orthotopic U87-MGvIII tumour model (Fig. 3). Five days post-cell implantation, the progression of intracranial malignancies was assessed by T_2_-weighed MRI (Fig. 3A). To confirm the targeting abilities of Z_EGFR:03115_, PET/CT studies were performed. Images were acquired at 1, 3, and 5 h post-administration using the well-established EGFR-targeting imaging agent ^18^F-AlF-NOTA-Z_EGFR:03115_ (Fig. S3A) (*n* = 3) [25]. The images recorded 3 h post-injection demonstrated preferential and focal accumulation of the radioconjugate in the tumour mass (Fig. 3A and Fig. S3A-B). Negligible activity was observed in the normal cerebral tissue that provided sharp delineation of the tumours with high tumour/parenchyma contrast. The ROI quantitative analysis showed a time-dependent increase of tumour radioactivity uptake with the highest value observed 5 h p.i. (%ID/g_50_ = 5.14 ± 1.17) (Fig. 3B). H&E staining of axial brain sections confirmed the presence of well-defined tumour masses, which were in line with PET/CT and MRI signals in vivo as well as radioactivity signals measured ex vivo (Fig. S3B). When Z_EGFR:03115_-IR700 was administered i.v., the strong fluorescence signal of the conjugate was detected ex vivo within the brain EGFR-positive lesions (2–3 mm in diameter) as early as 1 h post-injection in mice bearing MRI-confirmed tumours (Fig. 3C) (*n* = 3).

### Monitoring tumour response to Z_EGFR:03115_-IR700-PIT in vivo

**Fig. 4:**
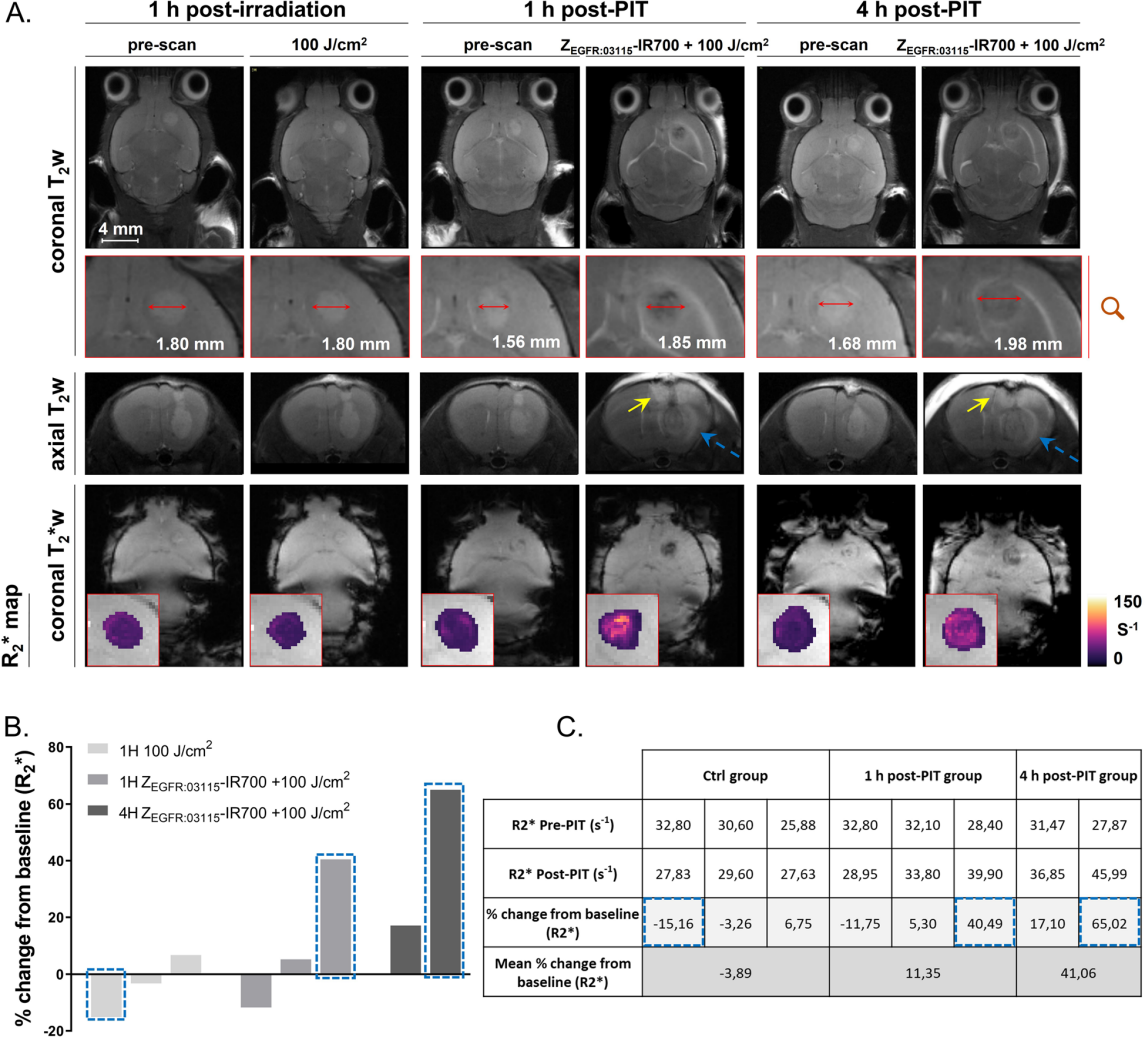
Early efficacy assessment using an MRI-based approach. **A** Coronal and axial T_2_-weighted, coronal T_2_*-weighted images (TE, 12 ms), and corresponding R_2_* maps in animals before and after 100 J/cm^2^ irradiation. Haemorrhagic areas can be seen 1 h after PIT on both T_2_-weighted and T_2_*-weighted images. Note the presence of cerebral oedema (yellow arrows). Blue arrows point out the tumour and peritumoural oedema. **B** Quantification of the R_2_* parameter in mice from the control group (100 J/cm^2^ light irradiation only, *n* = 3), 1 h (18 μg of Z_EGFR:03115_-IR700 + 100 J/cm^2^, *n* = 3) and 4 h post-PIT (18 μg of Z_EGFR:03115_-IR700 + 100 J/cm^2^, *n* = 2). Bars represent the % change of median R_2_* parameters from the baseline measured on ROIs drawn onto the tumours at TE = 3. **C** Table presenting the R_2_* values (s^−1^) of each mouse pre- and post-PIT with their corresponding % change from the baseline. R_2_* values from mice presented on the images are encircled with blue dashed lines

**Fig. 5:**
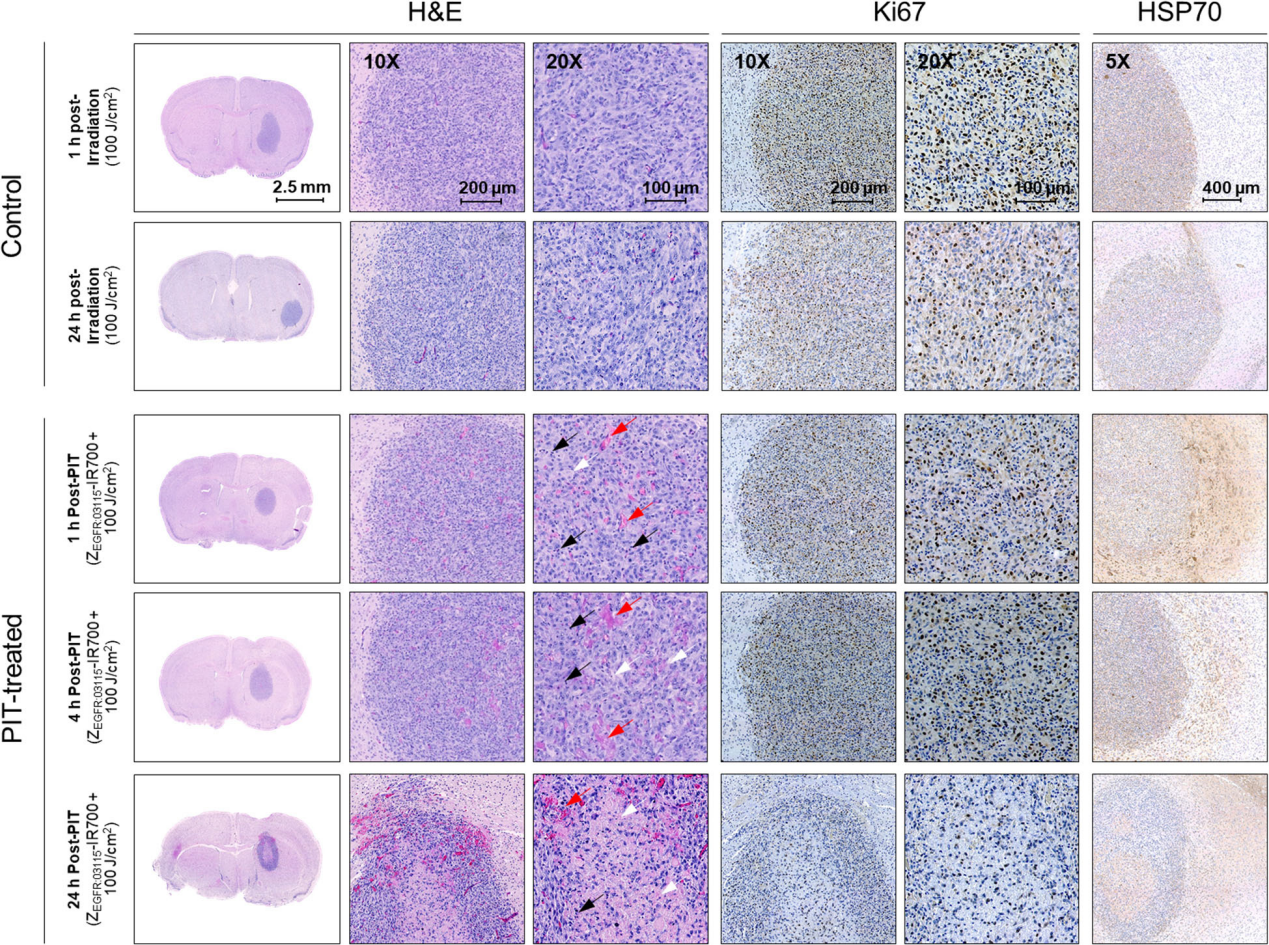
Histological analysis of the treatment efficacy in U87-MGvIII orthotopic tumours 1, 4, and 24 h post-PIT. Representative haematoxylin/eosin staining and corresponding Ki67 and HSP70 immunostaining of the brain tumours (× 10 and × 20). Red arrows indicate haemorrhagic areas, black arrows indicate pyknosis and apoptotic bodies, and white arrows indicate necrotic areas. Control mice were irradiated with 100 J/cm^2^. Treated mice were injected i.v. with 18 μg of the Z_EGFR:03115_-IR700 conjugate and irradiated with 100 J/cm^2^

To assess whether Z_EGFR:03115_-IR700-PIT shows an anti-tumour effect against GBM in vivo, subcutaneous and intracranial U87-MGvIII tumours were established in nude mice. In contrast to our previous studies using three Z_EGFR:03115_-IR700-PIT doses over three consecutive days [22], herein, we tested whether administration of just one dose would inhibit tumour growth. The conjugate (18 μg) was injected intravenously (Fig. S4A), and the subcutaneous tumours were irradiated 1 h later with 100 J/cm^2^. A significant delay in tumour growth was observed during the initial 7 days in the group receiving Z_EGFR:03115_-IR700-PIT compared to the control (light only 100 J/cm^2^) (Fig. S4B). However, due to the re-growth of measurable tumours at this point, a second PIT dose was delivered. Unfortunately, no further growth inhibition was observed (Fig. S4B). Next, we studied the early anti-tumour response to PIT using the orthotopic U87-MGvIII model by MRI. Following tumour establishment, T_2_- and T_2_*-weighted images were acquired at baseline and 1 or 4 h post-Z_EGFR:03115_-IR700-PIT (18 μg; 100 J/cm^2^). T_2_*-weighted imaging was selected because of its sensitivity to the presence of paramagnetic species such as deoxyhaemoglobin and its capability to detect cerebral and intratumoural micro-haemorrhage under pathologic conditions. T_2_*-weighted images showed an intratumoural signal decrease corresponding to haemorrhage and hemosiderin deposition 1 h post-NIR irradiation in mice treated with Z_EGFR:03115_-IR700-PIT as compared to the control group (Fig. 4A). We also observed an enlargement of the lesions on T_2_-weighted images caused by direct cytotoxic effects on tumour cells, damage to the tumour vasculature, and induction of an inflammatory reaction post-PIT, which resulted in the swelling of the surrounding brain tissue and a mass effect (Fig. 4A). Furthermore, increased signal intensity on T_2_-weighted images of parenchyma surrounding the tumour growing from the top of the skull along the pathway of cell implantation most likely corresponds to the cerebral oedema formation following PIT (Fig. 4A, yellow and blue arrows). The difference between areas of increased T_2_-weighted signal intensity in the surrounding tumour brain parenchyma (Fig. 4A, yellow vs blue arrows) was probably due to the changes in light distribution. The intratumoural quantification of R_2_* (Fig. 4B, C) indicated that mice exposed to Z_EGFR:03115_-IR700-PIT had a higher percentage of changes in R_2_* values compared to the controls (3.1% (*n* = 3) for the control group vs 11.3% (*n* = 3) and 41.1% (*n* = 2) for 1 h and 4 h groups post-PIT, respectively), which effectively confirmed an acute tumour response to PIT. Hypointense cores highlighted after PIT are compliant with early necrosis. These changes were consistent with H&E staining of the brain sections showing tumour necrosis and micro-haemorrhage patterns associated with pyknosis and apoptotic bodies on the margins of the PIT-treated tumours (Fig. 5, Fig. S4C). The tumour necrosis became extensive 24 h post-PIT, showing the strong effect of PIT especially on the core of U87-MGvIII tumours. Moreover, visual assessment of IHC staining for Ki67 indicated reduced cell proliferation 24 h post-conjugate irradiation. Our data also indicate that Z_EGFR:03115_-IR700-PIT triggered a substantial release of HSP70 within 1 h after treatment (Fig. 5).

### Z_EGFR:03115_-IR700 PIT triggers immune response in vivo

**Fig. 6:**
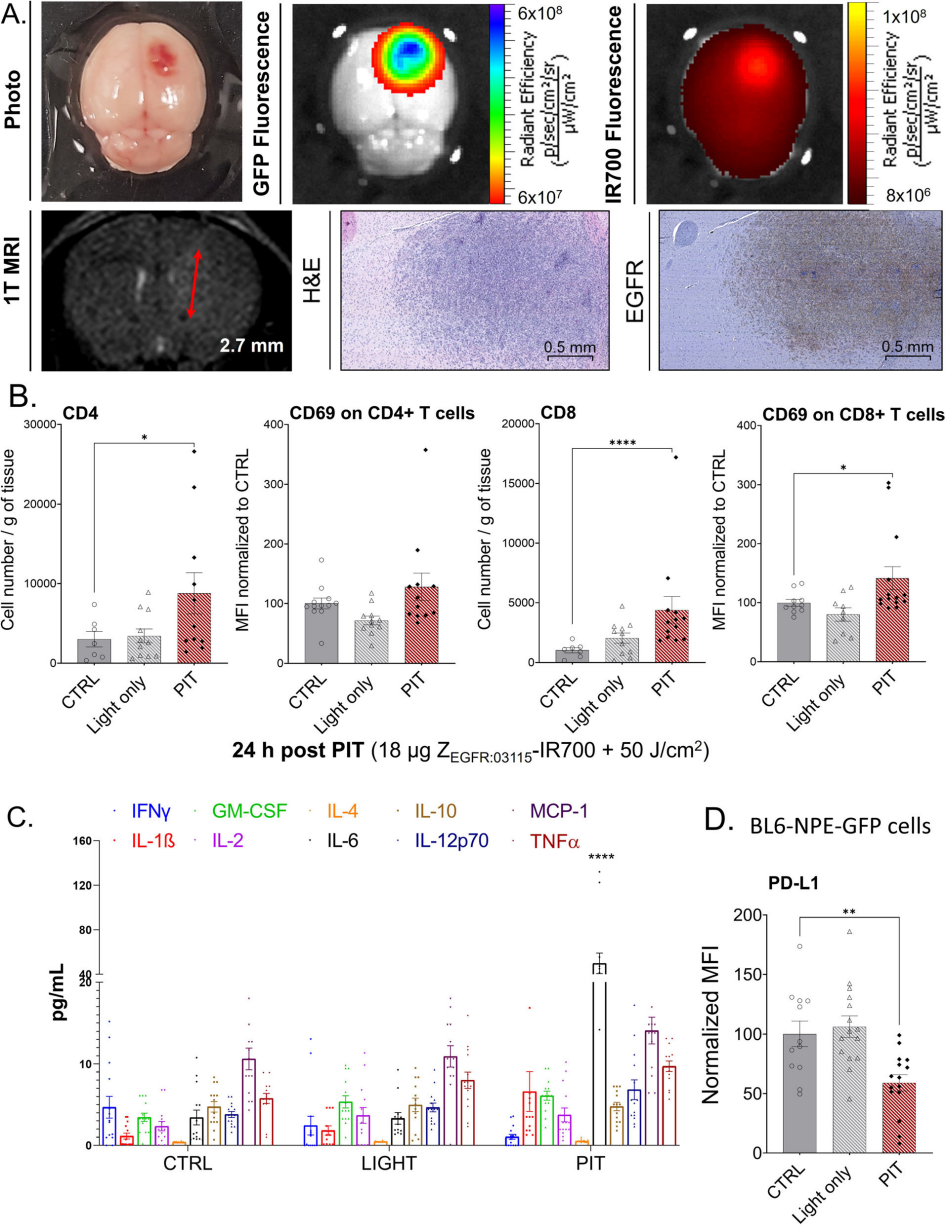
Characterisation of the syngeneic BL6-NPE-GFP-Luc orthotopic mouse model and immunologic response after PIT treatment. **A** In vivo axial T_2_-weighted MRI image acquired at 1 T and corresponding ex vivo photography and fluorescence image of the orthotropic BL6-NPE-GFP-Luc tumour 6 days after tumour cell engraftment. Fluorescence imaging and brain collection were performed 1 h after i.v. injection of 18 μg of Z_EGFR:03115_-IR700 (tumour diameter, 2.7 mm). Haematoxylin/eosin staining and EGFR immunostaining on the same mouse. **B** Quantification of intratumoural infiltration of CD4+ and CD8+ (gated on CD45+) T cells and their upregulation of CD69 early activation marker assessed by flow cytometry. Cells were isolated from brain tumour masses 24 h post-PIT treatment (18 μg Z_EGFR:03115_-IR700 + 50 J/cm^2^); *n* = 10 per group. Statistical difference between the PIT-treated and control cells was calculated using the Mann-Whitney *t* test. The results were considered significant when *p ≤ 0.05 and ****p ≤ 0.0001. **C** Mouse serum pro-inflammatory cytokine concentrations 24 h after PIT treatment (18 μg Z_EGFR:03115_-IR700 + 50 J/cm^2^). Statistical difference in comparison with the control group determined using ANOVA with Dunnett’s post hoc test. ****p ≤ 0.0001. **D** Normalised PD-L1 expression level on the surface of BL6-NPE-GFP-Luc cells (gated on GFP+) isolated from mouse brains 24 h post-PIT treatment (18 μg Z_EGFR:03115_-IR700 + 50 J/cm^2^) in comparison with the control groups (with or without 50 J/cm^2^ irradiation). Statistical difference in comparison with the control group determined using the Mann-Whitney *t* test. **p ≤ 0.01

We used the BL6-NPE-GFP-Luc syngeneic model to investigate whether a T cell-focused immune response can be elicited by light-activated Z_EGFR:03115_-IR700 in the brain setting (18 μg/50 J/cm^2^; Fig. 6A). In this case, we decided to lower the light intensity as we observed in some mice bearing intracranial U87-MGvIII tumours that the mass effect led to a significant deterioration of their condition. Flow cytometry analysis of tumour samples at 24 h post-PIT (18 μg/50 J/cm^2^) showed higher levels of CD4+ and CD8+ immune cells in tumours exposed to PIT compared to the control groups (Fig. 6B). The detailed gating strategy is implemented in Fig. S5A. A pronounced number of CD8+ cells was also detected on IHC slices of treated tumour (Fig. S5B). A similar but less pronounced trend was observed in CD69 expression level on T cells, an early activation antigen involved in the transmission of costimulatory signals (Fig. 6B). Thereafter, we measured the level of selected pro-inflammatory cytokines which have the ability to enhance the immune response towards tumours by activating NK cells, CD8+ T cells, and macrophages. We found a significant increase of IL-6 and an upward trend of IL-1β, TNF-α, and IL-12 levels from the control groups to the Z_EGFR:03115_-IR700-PIT group, while the level of IL-10, indicating immunosuppression revealed no change regardless of the treatment group (Fig. 6C).

Finally, to check the effect of PIT on the PD-1/PD-L1 axis, we assessed whether Z_EGFR:03115_-IR700-PIT induces changes in the PD-L1 expression on tumour cells by flow cytometry. We initially confirmed that IFN-γ stimulation leads to a considerable increase in PD-L1 expression in multiple GBM cell lines (Fig. S5C). Subsequently, we detected a significant decrease of PD-L1 in U87-MGvIII cells in response to Z_EGFR:03115_-IR700-PIT with and without IFN-γ stimulation compared to the controls in vitro (Fig. S5D-E). These results were next corroborated by in vivo findings showing a downregulation of PD-L1 also on the surface of BL6-NPE-GFP-Luc cells post-PIT (Fig. 6D).

## Discussion

Extensive GBM cell invasion into the normal brain parenchyma makes complete tumour removal practically impossible and disease recurrence inevitable. Besides, the GBM TME is recognised as highly immunosuppressive, posing a major hurdle for inducing immune-mediated destruction of remaining cancer cells. As a result, clinical trials evaluating checkpoint blockade in GBM patients have failed to demonstrate clear efficacy [9, 10]. Recently, it became clear that some treatment approaches can alert and trigger the immune response within the immunosuppressive GBM TME. For example, studies in preclinical models have shown that the combination of ICPIs with a concurrent administration of focal radiation therapy, cancer cell-directed immunotoxins, and oncolytic viruses increase anti-GBM immunity [29–31]. Moreover, EGFRvIII CAR-T cell therapy induced inflammatory responses in GBM patients turning “cold” GBM microenvironment into “hotter” without inducing neurotoxicity [32].

In the present study, we demonstrate that NIR-PIT may induce direct GBM cell killing via ICD and attracts T effector cells locally in the GBM TME. So far, mAbs-based conjugates have been most frequently utilised for PIT purposes [33, 34]. However, the large molecular size of mAbs and their extended blood circulation may slow penetration of the proteins into the tumour parenchyma. Consequently, it may hamper the response to PIT and result in long-lasting systemic photosensitivity [35].

To overcome such limitations, van Driela et. al. have recently demonstrated that the use of small EGFR-targeted nanobody-IRDye700DX conjugates (15 or 30 kDa) leads to higher tumour:background contrast and enhanced tumour necrosis when compared with full-size mAb-based IRDye700DX conjugate [36]. Along the same line, our previous studies suggested that affibody molecules (~ 7 kDa) conjugated to IR700 due to their rapid tumour accumulation and blood clearance are promising candidates for PIT purposes [22, 37].

Herein, we further demonstrated that Z_EGFR:03115_-IR700-PIT can trigger a local immune response in the brain tumour microenvironment. The conjugate binding to EGFR on the membrane of GBM cells induced receptor expression-dependent cell death upon NIR light exposure which was, in part, due to ROS production. Interestingly, Kato et al. have recently provided a theoretical mechanism by which photoactivated hydrolysis reaction following irradiation of mAb-based IR700 conjugates cause changes in the silicon-oxygen bond and silanol formation, which converts the dye from very hydrophilic to very hydrophobic [16]. Whether similar effects occur in response to irradiation of Z_EGFR:03115_-IR700 will need to be investigated. Additionally, the efficacy of Z_EGFR:03115_-IR700 in vitro increased in a conjugate concentration-dependent manner, and significant phototoxicity was observed within 1 h post-light exposure of conjugate-treated cells.

Of importance, several studies, including ours, provide evidence that PIT can induce mobilisation of DAMPs involved in ICD [28, 33, 37]. These molecules serve as an “eat-me” signal and mediate anti-tumour immune responses that are critical for the efficacy of the therapy and formation of long-term immunological memory [38, 39]. Therefore, we investigated whether irradiation of Z_EGFR:03115_-IR700 will result in the release of these danger signals. We observed high-level cell surface CRT exposure, rapid ATP secretion, and HMGB1 release only in PIT-treated cells, indicative of ICD. However, in cells treated with either Z_EGFR:03115_-IR700 or light alone, these signals were not enhanced compared to controls. Furthermore, significant release of DAMPs by PIT-treated GBM cells subsequently activated and promoted maturation of antigen-presenting iDCs, as indicated by a marked expression of CD86 and HLA-DR.

Thereafter, in order to determine whether the conjugate is capable of inducing selective tumour cell death in vivo, we treated mice bearing subcutaneous U87-MGvIII xenografts with Z_EGFR:03115_-IR700-PIT.

Burley et al. have recently reported that EGFR targeting affibody molecule (Z_EGFR:03115_) with high specificity recognise EGFR in vivo. For example, the U87-MGvIII-bearing mice injected with Z_EGFR:03115_-IR700 displayed a strong fluorescent signal as compared to Z_TAQ_-IR700 (a non-specific affibody molecule). The tumour fluorescent intensity of Z_EGFR:03115_-IR700 was 6-fold higher than Z_TAQ_-IR700 already 1 h post-injection [22]. Furthermore, when Z_EGFR:03115_ was radiolabelled with zirconium-89, only very low accumulation of the radioconjugate was found in tumours with low EGFR expression levels [25].

Apart from a targeting vector, also the light dose delivered and the method by which it is delivered are crucial to the success of PIT. However, physical dosimetry during PIT is a complex process due to the nature of dynamic interactions between light, conjugate, oxygen, and biological response of different tissues, which clearly depends on the concentration of cytotoxic photoproducts and on the intrinsic photosensitivity. In the murine models of GBM, the explicit dosimetry to map the distribution of light delivery and direct measurement of the light fluence are technically challenging. Therefore, for the purposes of this manuscript, we individually selected the intensity of light for U87-MGvIII and BL6-NPE-GFP-Luc models based on the initial validation experiments. For the xenograft model, the therapeutic light fluence was chosen to be 100 J/cm^2^ in order to maximise treatment efficacy considering the penetration of the NIR light and inevitable photobleaching of IR700 during the illumination. Of note, this light dose was reduced to 50 J/cm^2^ in the syngeneic model to lessen oedema-related swelling caused by direct cytotoxic effects on tumour cells and subsequent inflammation post-PIT. The irradiation of Z_EGFR:03115_-IR700 restrained the growth of subcutaneous U87-MGvIII tumours in the PIT-treated mice in comparison with controls (light only), which validated the model and procedure we employed.

Encouraged by this potent anticancer activity in vitro and in vivo, we further evaluated this approach in the brain setting. It is well known that GBM progression leads to blood-brain barrier (BBB) structural changes including neuronal death, astrocyte endfeet displacement, and heterogeneous pericyte and astrocyte subpopulations, all of which can reduce the barrier functions through the formation of fenestrations and disruption of tight junctions [40]. Even though it makes the BBB leaky and more permeable for small and large molecules, the barrier is still considered as one of the predominant restricting factors for the efficacy of therapies intended for the clinic. Given the limitations of planar optical imaging of brain tumours and quantification of fluorescence intensity, instead of Z_EGFR:03115_-IR700, we initially used the radiolabelled conjugate ^18^F-AlF-NOTA-Z_EGFR:03115_ to assess the efficacy of the affibody molecule in targeting EGFR-positive tumours in the brain setting. The acquired PET/CT images showed discrete focal accumulation of the radiotracer in the brain lesions already 1 h post-injection. Considering the small difference in size between the two conjugates, we expected Z_EGFR:03115_-IR700 to exhibit similar in vivo behaviour to ^18^F-AlF-NOTA-Z_EGFR:03115_.

Indeed, fluorescence images of the entire brain captured ex vivo post-Z_EGFR:03115_-IR700 administration clearly indicated accumulation of the conjugate in the tumour and provided insights into its delivery. Despite a relatively equal distribution of Z_EGFR:03115_-IR700 in the tumours, we observed some variability in the response to PIT between the mice. This could be linked to a non-uniform irradiation through the burr hole in the mouse skull resulting in uneven NIR-light delivery and light-induced photochemical production of ROS. In spite of these issues, hypointense signals were depicted on T_2_*w images of U87-MGvIII tumours within 1 h post-PIT that corresponded to microhaemorrhagic lesions. Moreover, histopathological examination of the brain sections revealed high levels of necrosis induced by irradiation of Z_EGFR:03115_-IR700 24 h post-treatment. Of importance, necrosis has been previously reported to be the characteristic form of cellular death post-PIT [41, 42]. Furthermore, cytoplasmic HSP70, a stress-inducible chaperone protein, was released from the cells as early as 1 h after Z_EGFR:03115_-IR700-PIT, as confirmed by IHC staining of tumour sections. As published earlier, the translocation of HSP70 depends on the NIR light dose and is related to either mitochondrial or direct surface stress disruption [43, 44]. Moreover, accumulating evidence suggests that HSP70 plays a role in DC maturation and activation of other antigen-presenting cells [45]. For example, it has been reported that HSP70 secreted from PDT-treated tumour cells promoted stimulation of DC and NK cells as well as the production of pro-inflammatory cytokines [46]. In addition, Korbelik et al. showed that HSP70 secreted post-PDT was captured by macrophages that triggered toll-like receptor-based signal transduction and production of TNFα [47]. Finally, we used the BL6-NPE-GFP-Luc syngeneic tumour model to look into the local immune response and activation of tumour-infiltrating lymphocytes post-Z_EGFR:03115_-IR700 PIT. Excitingly, we identified enhanced immunological response after conjugate irradiation which resulted in the attraction and activation of CD4+ and CD8+ T cells in PIT-treated tumours compared to the control group. Furthermore, the expression of both IL-1β and IL-6, which have the ability to enhance the immune response against tumours by activating CD8+ T cells was also markedly increased. Interestingly, we also observed that Z_EGFR:03115_-IR700-PIT reduced the level of compensatory immunosuppressive PD-L1 in U87-MGvIII and BL6-NPE-GFP-Luc cells in vitro. We speculate that the remaining PD-L1+ cells could still suppress the anti-tumour immune response and allow the tumour cells to survive immunologic cytotoxicity. Of note, Kleinovink et al. have recently shown in tumour models of colon carcinoma that the addition of CTLA-4 blockade prior to bremachlorin-PDT leads to a significant reduction in tumour burden compared to either treatment alone [48].

## Conclusions

In conclusion, the surgical options for GBM patients have not changed significantly over the last three decades and performing a complete tumour excision often presents an insuperable challenge. Residual tumour cells located in close proximity to critical functional areas are often left in the margins of the resection, leading to disease relapse. The possibility of enhanced surgical precision together with intra-operative adjuvant treatment could improve the outcome of GBM patients. In fact, it has been recently shown in patients with recurrent high-grade glioma that a combination of 5-ALA fluorescence-guided resection and open PDT after tumour removal is a promising strategy for local tumour control and targeting non-resectable, visibly fluorescent tumours [49]. Consistent with this, our studies highlight that Z_EGFR:03115_-IR700 fluorescence could guide resection of the tumour mass, and Z_EGFR:03115_-IR700-PIT lead to the eradication of residual tumour GBM cells simultaneously turning an immunosuppressive TME into an immune-vulnerable one.

Overall, more work is needed to fully unlock the potential of PIT as an effective treatment for GBM, especially concerning local and systemic immune responses and synergies with adjuvant treatments. In addition, there are practical aspects in the procedure that need further investigations including the assessment of light intensity, light delivery protocols, and dosimetry. In addition, as bleeding into the tumour may potentially result in oedema of brain parenchyma clinical application of PIT should be considered for GBM remnants within tumour resection cavity or for patients with small and deeply located tumours where stereotactic PIT could be attempted. Nevertheless, Z_EGFR:03115_–IR700-PIT holds a tremendous potential as a novel therapeutic approach against this aggressive type of brain tumour.

## Supplementary Information

**Additional file 1: Figure S1.** Characterisation of Z_EGFR:03115_-IR700 binding capacity on EGFR-positive GBM cells. **Figure S2.** Post-PIT DAMPs release. **Figure S3.** Ability of ^18^F-AlF-NOTA-Z_EGFR:03115_ to accumulate in orthotopic U87-MGvIII tumours. **Figure S4.** *In vivo* EGFR-targeted PIT in U87-MGvIII subcutaneous tumours. **Figure S5.** Immune response to PIT.

## Data Availability

The datasets used and analysed during the current study are available from the corresponding authors on reasonable request.

## Abbreviations

5-ALA: 5-Aminolevulinic acid
ATP: Adenosine triphosphate
BBB: Blood-brain barrier
CRT: Calreticulin
DAMPs: Damage-associated molecular patterns
DC: Dendritic cell
DNA: Deoxyribonucleic acid
EGFR: Epithelial growth factor receptor
GBM: Glioblastoma
HMGB1: High-mobility group box-1
HSP70/90: Heat shock protein 70/90
ICD: Immunogenic cell death
iDCs: Immature dendritic cells
MHC: Major histocompatibility complex
NAC: *N*-acetyl-l-cysteine
NIR-PIT: Near infrared-photoimmunotherapy
NK cells: Natural killer cells
PD-L1: Programmed death-ligand 1
PDT: Photodynamic therapy
PIT: Photoimmunotherapy
ROS: Reactive oxygen species
SOSG: Singlet oxygen sensor green reagent
TME: Tumour microenvironment
