# Correction: Triggering anti-GBM immune response with EGFR-mediated photoimmunotherapy

**DOI:** 10.1186/s12916-022-02388-z

**Published:** 2022-05-03

**Authors:** Justyna Mączyńska, Florian Raes, Chiara Da Pieve, Stephen Turnock, Jessica K. R. Boult, Julia Hoebart, Marcin Niedbala, Simon P. Robinson, Kevin J. Harrington, Wojciech Kaspera, Gabriela Kramer-Marek

**Affiliations:** 1 Division of Radiotherapy and Imaging, The Institute of Cancer Research, 123 Old Brompton Road, London, SW7 3RP UK; 2 Department of Neurosurgery, Medical University of Silesia, Regional Hospital, 41-200 Sosnowiec, Poland

**Correction: BMC Med 20, 1-17 (2021)**

**https://doi.org/10.1186/s12916-021-02213-z**

The original article [1] contained an erroneous grant number. The grant number has since been corrected.
