# Supplementary material for: Triggering anti-GBM immune response with EGFR-mediated photoimmunotherapy
Source: BMC Med. 2022 Jan 21;20:16. doi: 10.1186/s12916-021-02213-z (PMC8780306; doi:10.1186/s12916-021-02213-z)
Supplement: Supplementary file 1 — Additional file 1: Figure S1. Characterisation of ZEGFR:03115-IR700 binding capacity on EGFR-positive GBM cells. Figure S2. Post-PIT DAMPs release. Figure S3. Ability of 18F-AlF-NOTA-ZEGFR:03115 to accumulate in orthotopic U87-MGvIII tumours. Figure S4. In vivo EGFR-targeted PIT in U87-MGvIII subcutaneous tumours. Figure S5. Immune response to PIT. [file 12916_2021_2213_MOESM1_ESM.docx]

**Supplementary Materials**

**Title: Triggering anti-GBM immune response with EGFR-mediated photoimmunotherapy**

**Authors:** Justyna Mączyńska^1#^, Florian Raes^1#^, Chiara Da Pieve^1^, Stephen Turnock^1^, Jessica K.R. Boult^1^, Julia Hoebart^1^, Marcin Niedbala^2^, Simon P. Robinson^1^, Kevin J. Harrington^1^, Wojciech Kaspera^2*^, Gabriela Kramer-Marek^1*^

^#^Justyna Mączyńska and Florian Raes contributed equally.

**Authors’ Affiliations:**

^1^Division of Radiotherapy and Imaging, The Institute of Cancer Research, London, UK

^2^Department of Neurosurgery, Medical University of Silesia, Regional Hospital, Sosnowiec, Poland

**Contents:**

Materials and Methods

Figure S1. Characterisation of Z_EGFR:03115_-IR700 binding capacity on EGFR-positive GBM cells.

Figure S2. Post-PIT DAMPs release.

Figure S3. Ability of ^18^F-AlF-NOTA-Z_EGFR:03115_ to accumulate in orthotopic U87-MGvIII tumours.

Figure S4. *In vivo* EGFR-targeted PIT in U87-MGvIII subcutaneous tumours.

Figure S5. Immune response to PIT.

**Materials and Methods**

**Preparation of Z_EGFR:03115_-IR700**

Z_EGFR:03115_-Cys (250 μg, 37.2 nmol, AffibodyAB, Sweden) was incubated with Tris(2-carboxyethyl)phosphine (TCEP) (10 mg/mL of TCEP in 1 M phosphate buffer, pH 7.3, Thermo Fisher Scientific, UK) (930 nmol, 25-fold molar excess) for 5 min at 85°C and then at room temperature for 25 min using a ThermoMixer® (500 rpm; Eppendorf, UK). A freshly prepared 10 µM solution of IRDye700DX-maleimide (IR700, LI-COR® Bioscience, US) in water was then added to the mixture (190 nmol, 5-fold molar excess) and the reaction mixture was incubated at 40°C at 500 rpm. After 2 h the solution was kept on ice for 10 min and centrifuged at 10000 rpm for 5 min. The supernatant was collected and purified by Zeba™ Spin Desalting Column (7K MWCO, 2 mL, Thermo Fisher Scientific, UK) equilibrated in PBS following the manufacturer’s instructions. The pellet was re-suspended in PBS and combined to the eluate from the desalting column. The concentration of the affibody-dye conjugate was determined by UV-vis spectroscopy using a NanoDrop™ 2000 Spectrophotometer (Thermo Fisher Scientific, UK) at 689 nm. The yield of Z_EGFR:03115_-IR700 conjugation was found to be 65.6 ± 3.0%. SDS-PAGE and Pierce Silver Stain kit (Thermo Fisher Scientific, UK) were used to confirm the purity of the conjugate. The protein bands were visualised on a ChemiDoc™ XRS+ Imager (Bio-Rad, UK) using Image Lab™ 6.0 (Bio-Rad, UK) software. Corresponding fluorescence bands were imaged on a Typhoon™ FLA7000 imager (ex. filter: 635 nm; em. filter: 670 nm, GE Healthcare Life Science, UK) and a IVIS Spectrum/CT (ex. filter: 675 nm, em. filter: 720 nm).

**Cell lines and cell culture**

Human glioblastoma cell line DKMG and murine GBM cell line GL261 were purchased from the Celther Polska (Poland) and the German Collection of Microorganisms and Cell Cultures (DSMZ, Germany), respectively. U87-MG and U87-MGvIII were kindly provided by Dr Frank Furnari (Ludwig Cancer Research, US) and maintained as previously described ([18](#_ENREF_18)). The cells were cultured as monolayers in either DMEM ( U87-MG; Thermo Fisher Scientific, US), DMEM containing 400 g/mL G418 (U87-MGvIII; Thermo Fisher Scientific, US), or RPMI-1640 (DKMG, GL261; Thermo Fisher Scientific, US), all supplemented with 10% heat-inactivated fetal bovine serum (FBS; Thermo Fisher Scientific, US) at 37°C in a humidified atmosphere containing 5% CO_2_.

The primary, patient-derived cell lines WSz4, WSz50, WSz57 have been recently established in our lab and were grown as described elsewhere ([17](#_ENREF_17)). Briefly, cells were cultured on laminin-coated (Merck, Germany) flasks under stem cell conditions in DMEM/Nutrient Mixture F-12/Ham medium (Sigma, UK) supplemented with N2 and B27 (Thermo Fisher Scientific, US), 2 μg/mL Heparin (StemCell Technologies, UK), GlutaMAX™, antibiotic-antimycotic solution (Life Technologies, UK) and 20 ng/mL each of recombinant human growth factors bFGF and EGF (both from StemCell Technologies, UK). BL6-NPE-GFP-Luc murine glioblastoma cell line was kindly provided by Dr Steven Pollard (University of Edinburgh, UK). Cells were cultured in DMEM/Nutrient Mixture F-12/Ham medium supplemented with glucose, MEM NEAA, BSA, 2-mercaptoethanol, N2 and B27 (all from Thermo Fisher Scientific, US), mouse EGF, human FGF (both from PeproTech, US) and laminin (Cultrex® 3D Culture Matrix Laminin I, Trevigen, US).

The genetic origin of cells was tested and authenticated by short tandem repeat (STR) DNA profiling analysis (Eurofins Medigenomix, Germany). The cells were also routinely tested and found to be negative for Mycoplasma contamination (PCR detection kit, Surrey Diagnostics Ltd, UK).

**Singlet oxygen production assay**

Singlet oxygen (^1^O_2_) production was determined using the Singlet Oxygen Sensor Green reagent (SOSG, Thermo Fisher Scientific, UK) according to the protocol provided by the manufacturer. Briefly, medium alone, SOSG alone, Z_EGFR:03115_-IR700 (0.25 μM) and, Z_EGFR:03115_-IR700 (0.25 μM) plus SOSG were irradiated with the LED 690-66-60 (Marubeni, Germany) from 0 s (0 J/cm^2^) up to 30 min (22.5 J/cm^2^). The SOSG fluorescence intensity post-irradiation was measured using an IVIS Spectrum/CT imaging system (PerkinElmer, UK) (ex. filter: 465 nm, em. filter: 520 nm; 1 s integration time). Data are represented as mean + SD (n = 3 independent experiments).

**Cellular binding of Z_EGFR:03115_-IR700**

Human and murine GBM cells (3 × 10^5^) were harvested and incubated in medium with Z_EGFR:03115_-IR700 (30 nM) for 1 h at 4°C. Samples were washed twice with cold PBS post-incubation and analysed using flow cytometry (BD™ LSRII; ex. 635 / em. 730/45).

To test the targeting specificity and internalisation of the conjugate, cells (2 × 10^5^) were plated on confocal glass-bottomed dishes (Thermo Fisher Scientific, US) in a complete medium with Z_EGFR:03115_-IR700 (1 µM) for 1 h at 4°C or 1, 3 and 6 h at 37°C. Hoechst®33342 (nuclear stain; 5 µg/mL; Thermo Fisher Scientific, UK) was used for nuclear counterstaining 30 min before imaging. PBS-washed samples were imaged with a Zeiss LSM700 confocal microscope (Carl Zeiss Inc, Germany) equipped with 405 nm and 639 nm lasers and appropriate filters. Data were analysed using Zen2009 software (Carl Zeiss Inc, Germany).

***In vitro* PIT studies**

U87-MGvIII cells were grown as monolayers seeded (3 × 10^5^) on 35 mm in diameter petri dishes 24 h before experiments. Afterwards, cells were incubated with Z_EGFR:03115_-IR700 (0.1 to 1 µM) for 1, 3 or 6 h at 37°C. Media were then changed for phenol red-free DMEM medium and cells were irradiated (8 or 16 J/cm^2^) using a LED light source (L690−66−60, Marubeni America Co., US) at the wavelength of 690 ± 20 nm (peak mean power density of 12.5 mW/cm^2^, current of 285 mA).

Cell viability was determined using the CellTiter-Glo® (Promega, US) luminescent assay 24 h post-light exposure. To assess ROS production, 5 µM 2',7'-dichlorofluorescein diacetate (DCFDA; Sigma, UK) was added to phenol-red free medium during irradiation. N-acetylcysteine (NAC; Sigma, UK) was used as an antioxidant to inhibit ROS generation. Tert-Butyl Hydrogen Peroxide (TBHP) was used as a positive control. Immediately following treatment, the cells were collected and the DCFDA fluorescence was measured according to the manufacturer’s protocol using a flow cytometer (ex. 488 nm, em. 530/30 nm). To perform the Annexin V/PI assay, the cells were dissociated with trypsin (Thermo Fisher Scientific, US) at 1, 4, and 24 h post-irradiation and suspended in 0.1 mL of propidium iodide (PI; 0.5 μg/mL) and Annexin V-Alexa Fluor® 488 (Annexin V/Dead Cell Apoptosis Kit, Thermo Fisher Scientific, UK) according to the manufacturer's instruction. Stained cells were analysed using flow cytometry (AnnexinV-AlexaFluor488™ ex. 488 nm, em. 530/30 nm, PI ex. 488 nm, em. 610/20 nm). For analysis, the cells were subdivided into the following populations: viable (Annexin V-/PI-), apoptotic (Annexin V+/PI-), and late apoptotic/necrotic (Annexin V+/PI+) cells.

To determine post-PIT DAMP release (ATP and HMGB1), the cell medium was collected at 5 min, 1, 4, 8, and 24 h post-light irradiation and centrifuged at 500×g for 5 min at 4°C. The supernatants were used to determine the extracellular ATP using the ENLITEN® ATP assay system (Promega, US) and HMGB1 using an HMGB1 ELISA kit (Tecan, IBL International, Germany) following the manufacturers’ guidelines.

**Co-culture with dendritic cells**

Firstly, peripheral blood mononuclear cells (PBMCs) were isolated from leukocyte cones (NC24, obtained from healthy donors and supplied by the NHS blood and transplant service, UK), by gradient centrifugation using Ficoll^®^ Paque Plus (Merck, Germany). CD14+ monocytes were then separated from the PBMCs via immunomagnetic negative selection using an EasySep™ Direct Human Monocyte Isolation Kit (StemCell Technologies, UK). To promote the differentiation of CD14+ monocytes into immature dendritic cells (iDCs) in culture, RPMI medium was supplemented with FBS, interleukin-4 (IL-4, 500 U/mL; PeproTech, US) and granulocyte-macrophage colony-stimulating factor (GM-CSF, 800 U/ml; PeproTech, US) for 5 days. Next, the iDCs were co-cultured with PIT-treated U87-MGvIII cells at an iDC/cancer cell ratio of 1:2. iDCs stimulated by E.coli lipopolysaccharide (LPS, 100 ng/mL; Merck, Germany) for 12 h were used as a positive control. After 48 h, all floating cells were collected and stained with Pacific Blue™ anti-human CD14, PE anti-human CD11c, FITC anti-human CD86, AlexaFluor® 700 anti-human CD40 and APC anti-human HLA-DR (all antibodies from BioLegend, US) and PI. Flow cytometry was performed on a BD™LSRII machine. DC-maturation markers were assessed on alive (PI-), CD14- and CD11c+-gated cells populations using FlowJo software.

**Western blot**

Antibodies against EGFR (Catalogue No 2646; 1:1000), , HSP70 (4872; 1:500), HSP90 (4877; 1:1000), Calreticulin (12238; 1:1000), HMGB1 (3935; 1:250), β-actin (4967, 1:1000), GAPDH (5174; 1:1000) and respective secondary anti-rabbit or anti-mouse HRP-conjugated antibodies were used. All antibodies were purchased from Cell Signalling Technology, UK. Western Blot membranes were scanned using a ChemiDoc™ XRS+ System (Bio-Rad, UK). Densitometric analysis was performed for the EGFR, CRT, HMGB1, HSP70 and HSP90, relative to the loading control, using ImageJ Fiji package (version 1.53c, NIH, USA). Expression levels were normalized to each control sample. Data are represented as mean + SEM (n = 3 independent experiments).

**Animal care**

All experiments were performed in compliance with licences issued under the UK Animals (Scientific Procedures) Act of 1986, and following local ethical review. Studies were compliant with the UK National Cancer Research Institute Guidelines for Animal Welfare in Cancer Research and the ARRIVE (animal research: reporting *in vivo* experiments) guidelines. All procedures were carried out under an approved Home Office project license (number PPL PCC916B22). Female NCr athymic mice (6-8 weeks old) obtained from the in-house breeding colony were used. Female C57BL6/6J mice mice (7-8 weeks old) were obtained from Charles River Laboratories. Animals were housed in 500 square cm Allentown Nexgen cages and given access to mice maintenance food (Labdiet, US) and water ad libitum. Environmental conditions were a temperature of 21^o^C±1^o^C, humidity of 55%±10%, and a 12:12 light:dark cycle with lights. Environmental enrichment included bedding substrate of Corn Cob 6/8, Aspen chew stick 5x1x1 cm (Datesand, product code CS3C15) and one Bed r’nest paper wool nesting material (Datesand, Product code BEDRNEST).

**Mouse models**

Before implantation of orthotopic GBM tumours, mice were anaesthetised with an IP injectable ketamine/xylazine mixture. Following each mouse was stereotactically injected with 3 μL of either human U87-MGvIII (1 × 10^5^) or murine BL6-NPE-GFP-Luc (3 × 10^5^) cells in PBS. The injection site was located 2 mm lateral to the sagittal suture, 1 mm anterior to the coronal suture, and 2.5 mm deep from the surface of the skull. Mice were monitored daily for any adverse side-effects and 1T MRI images were acquired 5−6 days post-implantation to confirm tumour presence. For the subcutaneous GBM xenografts, U87-MGvIII cells (5 × 10^5^) re-suspended in 100 μL of 7:3 v/v mixture of PBS and Matrigel™ (Matrigel^TM^ Matrix, BD Bioscience) were injected into the right shoulder. Once tumours reached approximately 60 mm^3^, mice were randomly distributed into the experimental groups. Once tumours reached approximately ~60 mm^3^, mice were randomly distributed into experimental groups. During treatment, animals were monitored daily and the tumour volume was assessed via calliper measurements every other day and calculated via the formula V=1/2(length × width ×height). When volumes reached 500 mm^3^, mice were sacrificed and tumours collected for *ex vivo* analysis.

**PIT *in vivo***

For PIT treatment studies, subcutaneous GBM xenografts (n = 3-5 per group) were randomised into the following treatment groups: (i) light exposure only (100 J/cm^2^); (ii) 18 µg Z_EGFR:03115_-IR700 i.v. with light exposure (100 J/cm^2^). For immunocompetent mice, 50 J/cm^2^ light dose was used. With the surrounding normal tissue covered with a photon opaque material, the tumours were irradiated with a LED light source (L690−66−60, peak 690 ± 20 nm) 1 h post-conjugate injection. The optical fluence of 100 J/cm^2^ was implemented with a continuous illumination and a fixed current intensity of 300 mA for 516 s. There was approximately 2−3 mm distance between tumour and the source of light. The delivered energy was previously characterised by an optical power meter (PM160T, ThorLabs, US). In orthotopic GBM xenografts, the distance between the mouse skull and the source of light was approximately 4−5 mm, and the irradiation time was either 564 or 1128 s for 50 J/cm^2^ or 100 J/cm^2^, respectively. Mice were given 2 mg/kg of dexamethasone phosphate (Dexaven®, Pharmaswiss, Czech Republic) prior and 12 h post-irradiation.

**MR imaging**

To monitor orthotopic tumour growth, mice were imaged under isoflurane anaesthesia using the 1T M3™ MRI system (Aspect Imaging, Israel) with a T_2_-weighted pulse imaging sequence and a dedicated head coil (TR 3000 ms, TE 77.9 ms, TI 100 ms, 20 averages, flip angle 90°, resolution of 156 x 155 µm, 1mm thickness). Images were then processed with RadiAnt DICOM Viewer v4.6 (Medixant, Poland). To perform high-resolution acquisitions, mice were imaged under isoflurane anaesthesia using the 7T Biospec® horizontal micro-imaging system (Biospec® 70/20, Bruker, US) with a 2 × 2 cm brain array coil (TR = 4500 ms, TE = 36 ms, 1 average, RARE factor = 8, 98 × 98 μm resolution, 1 mm thickness). The measurement of the baseline transverse relaxation rate R_2_ (s^-1^), which is sensitive to the concentration of paramagnetic species (e.g. deoxyhaemoglobin) was quantified using a multiple gradient-recall echo (MGE) sequences (TR = 200 ms, 8 TE = 3-24 ms spaced 3 ms apart, 8 averages, 156 x 156 µm in-plane resolution, 1 mm slice thickness). Tumour R_2_* maps were calculated from regions of interest drawn for each tumour-containing slice from the MGE images acquired prior to and following light irradiation by fitting a single exponential to the signal intensity echo time curve on a voxel-by-voxel basis using a robust Bayesian approach processed by in-house software (ImageView, developed in IDL; ITT Visual Information Systems, US).

**PET imaging**

Mice (n = 5) with MRI-confirmed brain tumours received an i.v. injection of ^18^F-AlF-NOTA-Z_EGFR:03115_ (12 μg; 2.4 ± 0.15 MBq/mouse). For PET/CT scans mice were anesthetized with isoflurane (1.5% in air) and images acquired 1, 3, and 5 h post-injection of the radioconjugate using an Albira PET/SPECT/CT system. Whole body 10 min static images were acquired with a 358 to 664 keV energy window, followed by CT acquisition. Scatter and attenuation corrections were applied using their respective CT scans. The PET images were reconstructed using a maximum-likelihood expectation-maximization (MLEM) algorithm (12 iterations) with a voxel size of 0.5 × 0.5 × 0.5 mm^3^. High-resolution CT scans were performed with the X-ray tube set-up at a voltage of 45 kV, current of 400 µA, 250 projections (1 s per projection), and a voxel size of 0.5 × 0.5 × 0.5 mm^3^. The CT images were reconstructed using a filtered-back-projection (FBP) algorithm. Image analysis was performed using the PMOD software package (PMOD Technologies Ltd., Switzerland). The mean counts (PET Mean) and the PET Peak (mean of the 50 voxels with highest counts within the tumour volume) were recorded per mouse and converted into the percentage of the injected dose per gram of tissue (%ID/g) using a calibration factor (MBq/g/counts) determined from scanning a ^18^F source of known activity and volume.

**Autoradiography**

Dissected tumour and brain tissue samples were collected and immediately embedded in an optimal cutting temperature compound (Tissue-Tek® O.C.T, Netherlands) and snap-frozen in liquid nitrogen. Embedded tumours were sectioned with a cryostat (Shandon Fe Cryostat, Thermo Fisher Scientific, UK) into 10 μm thick slices and mounted on microscope slides. Freshly cut tumour sections were placed for 2 h in contact with a phosphor film and read using the Typhoon™ FLA 7000 imager (ex. filter: 650nm; em. filter: 390 nm; 25 μm pixel size; GE Healthcare, US).

**Fluorescent imaging**

*In vivo* and *ex vivo* fluorescence images were acquired with an IVIS Spectrum/CT (Perkin Elmer, US), generating a pseudo-coloured image representing light intensity, and superimposed over a greyscale reference image (ex. 675 nm, em. 720 nm). Mice anesthetised by 1.5% isoflurane were placed on a thermostatically controlled heating pad (37°C) during imaging. Acquisition binning and duration were set depending on tumour fluorescence.

**Immunohistochemistry**

Formalin-fixed brain and tumour tissues were embedded in paraffin, sectioned (5 μm-thick slices), and mounted on microscope slides. Frozen embedded tissues were sectioned into 10 μm-thick slices, and mounted on microscope slides before being fixed in ice-cold acetone. Multiple sections were taken at regular intervals across each brain and tumour, with sequential sections being stained with H&E (Leica Biosystems, Germany), anti-Ki67 mAb (1:400, Cell Signalling Technology, US) anti-EGFR mAb (1:400, Dako, UK), and anti-HSP70 (1:500, Santa Cruz Biotechnology, US). Heat-induced epitope retrieval of sections was performed for 3 min in a pressure cooker containing citrate-based antigen unmasking solution (pH 6.0; Vector Laboratories, US). Incubation with the primary antibodies was performed overnight (4°C), followed by incubation with HRP linked anti-mouse or anti-rabbit secondary antibodies (1:500, Cell Signalling Technology, US) for 1 h at RT. Chromogen development was performed using the ImmpactDAB assay kit (Vector Laboratories, US), and sections were counterstained with Gills III haematoxylin (Merck, Germany) before mounting. Slides were scanned with the NanoZoomer-XR (Hamamatsu Photonics, Japan) for digital processing.

**Tumour, T cells isolation and gating strategy**

Tumour and surrounding brain tissue were harvested and dissociated via enzymatic digestion (Liberase TL, Roche, Switzerland). Single-cell suspension was prepared by straining the digested tissue through a 70 µm mesh. Fc receptor-blocked (TruStain FcX™ anti-mouse CD16/32, Biolegend) cells were stained with anti-CD8a-BV421, anti-CD45-Alexa Fluor® 700, anti- PD-L1-PE (all from Biolegend, US), anti-CD4-BV480, anti-CD69-BB700 (both from BD, US), and a fixable viability dye eFluor780 (Thermo Fisher Scientific, US) for 20 min at 4°C. Cells were fixed with 2% paraformaldehyde (Merck, Germany) and stored at 4°C until the flow cytometric analysis. Data were analysed using FlowJo software (FlowJo LLC, US) Gates for positive events were set via Fluorescence Minus One (FMO) controls. The flow cytometry gating strategy for T cells was implemented by staining individual cells extracted from whole tumor tissues for the T cell markers CD4, CD8, CD45 and the activation marker CD69. Firstly, a forward scatter height vs. forward scatter area density plot was used to exclude doublets. Lymphocytes were then identified by a low forward scatter and low side scatter gate. CD4+ and CD8+ T cells were then identified within a CD45+ gate, and CD69 expression on activated T cells identified within the CD4+ and CD8+ gate.

**Statistical analysis**

Statistical significance for the *in vivo* data was determined using an unpaired t test with multiple comparisons using the Holm-Sidak method. Three to five experimental replicates were performed for the *in vitro* data. Statistical significance was determined using Mann-Whitney *t* test or ANOVA with Dunnett’s *post hoc* test for comparison of more than 2 groups. All analyses were processed with Prism software (GraphPad Software v7.0). Statistical differences were considered significant if p ≤ 0.05.

**
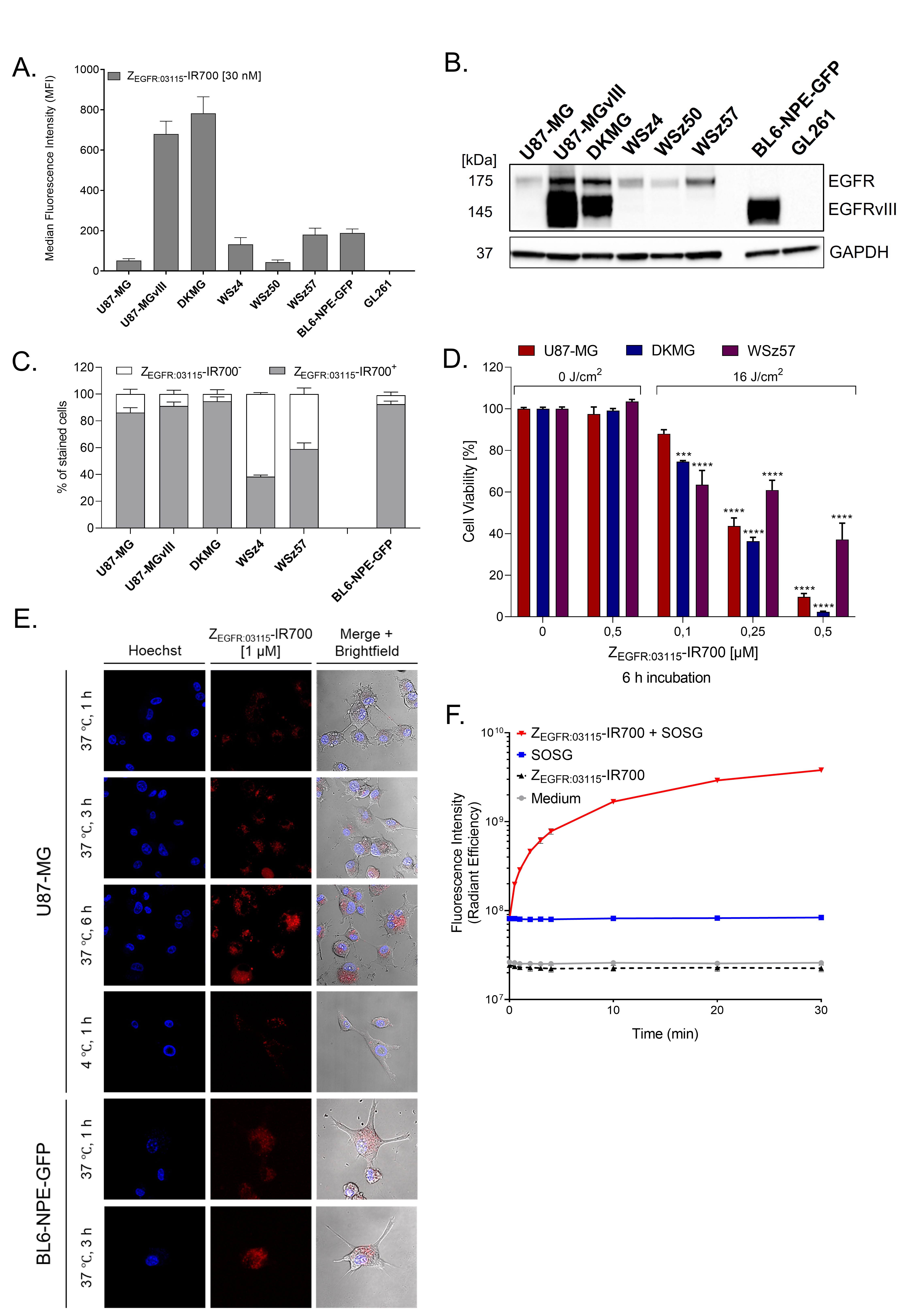
**

**Figure S1. Characterisation of Z_EGFR:03115_-IR700 binding capacity on EGFR-positive GBM cells. (A)** Z_EGFR:03115_-IR700 (30 nM) binding (1 h, 4°C) assessed by flow cytometry. Data are presented as mean ± SEM (n=3). **(B)** EGFR and EGFRvIII expression levels detected by Western blot in a panel of cancer cell lines. GAPDH was used as loading control. Protein loading 10 µg/well. **(C)** Percentage of positively stained cells (*grey bar*) with Z_EGFR:03115_-IR700 (30 nM) within live cell population assessed by flow cytometry. Data are presented as mean ± SEM (n≥4)**. (D)** Decrease in cell viability as assessed by the CellTiter-Glo® luminescent cell viability assay 24 h post-treatment in GBM cells (U87-MG, DKMG and WSz57) following 6 h incubation with the Z_EGFR:03115_-IR700 (0-0.5 μM) and irradiation with 16 J/cm^2^. The results were normalised to the control cells. Data are presented as mean ± SEM (n=3). Statistical difference in comparison to control (untreated) group determined using ANOVA with Dunnett’s *post hoc* test. ****p≤0.0001, ***p≤0.001. **(E)** Location and internalization of Z_EGFR:03115_-IR700 (1 μM; red) in human U87-MG and murine BL6-NPE-GFP GBM cells after 1, 3 or 6 h of incubation at 4 or 37°C imaged with a confocal microscope. Hoechst (cell nuclei; blue) was used as counterstaining. **(F)** Singlet oxygen (^1^O_2_) generation by NIR light-activated Z_EGFR:03115_-IR700 (0.25 μM) determined as amplified Singlet Oxygen Sensor Green reagent (SOSG) fluorescence intensity in cell-free conditions. Data are presented as mean ± SEM (n=3).


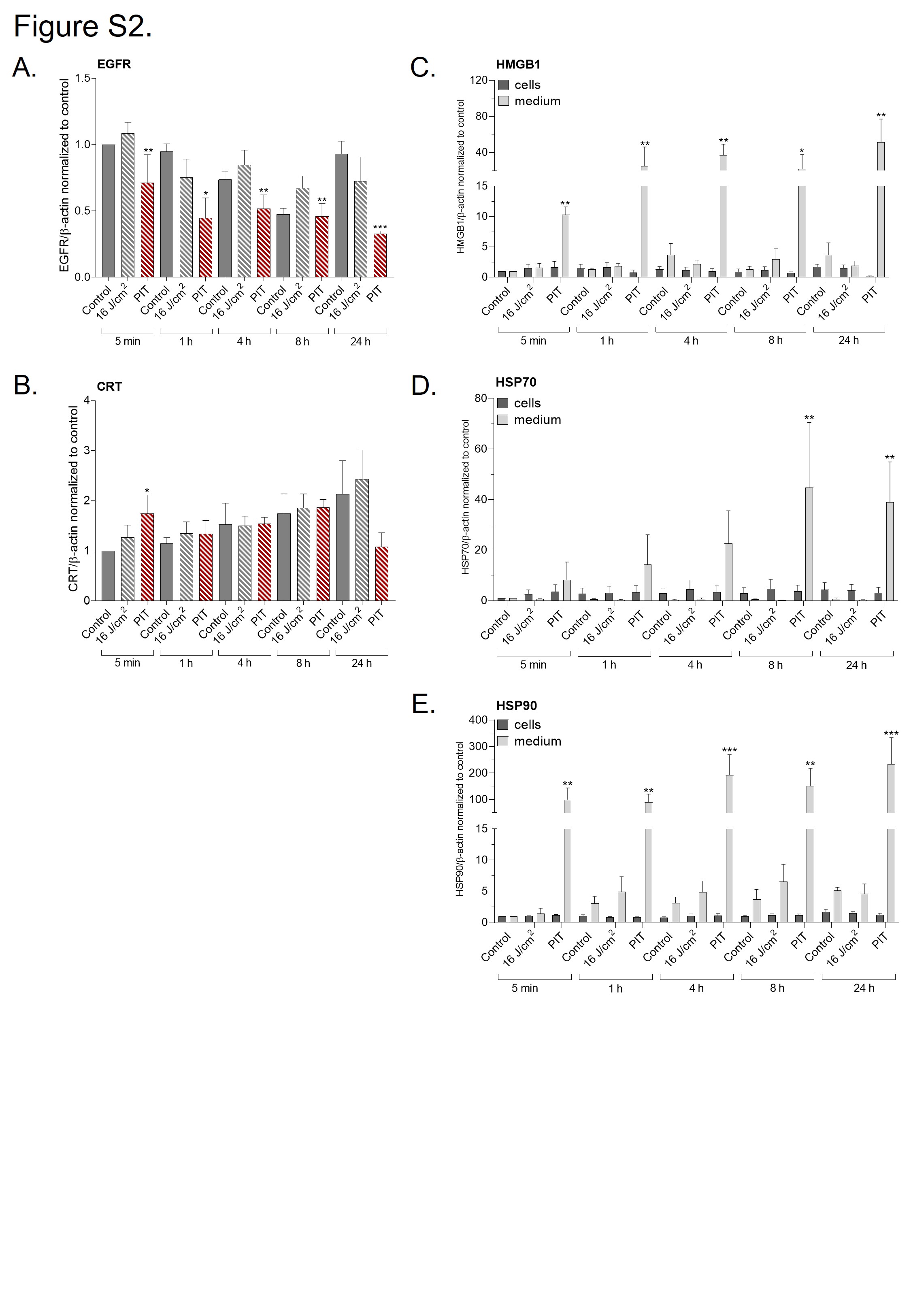


**Figure S2. Post-PIT DAMPs release.** Densitometric analysis of the optical density of **(A)** EGFR, **(B)** CRT, **(C)** HMGB1, **(D)** HSP70 and **(E)** HSP90 proteins bands in cells and cell supernatants (medium) of the U87-MGvIII line depending on time after treatment (0.25 μM of Z_EGFR:03115_–IR700 + 16 J/cm^2^) compared to light exposed (16 J/cm^2^) and control cells. The results are presented as the ratio of the test protein to the quantitative control (β-actin). Data are presented as mean ± SEM (n=3). Statistical difference in comparison to the control group determined using ANOVA Kruskal-Wallis test. ***p≤0.001, **p≤0.01, *p≤0.05.

**
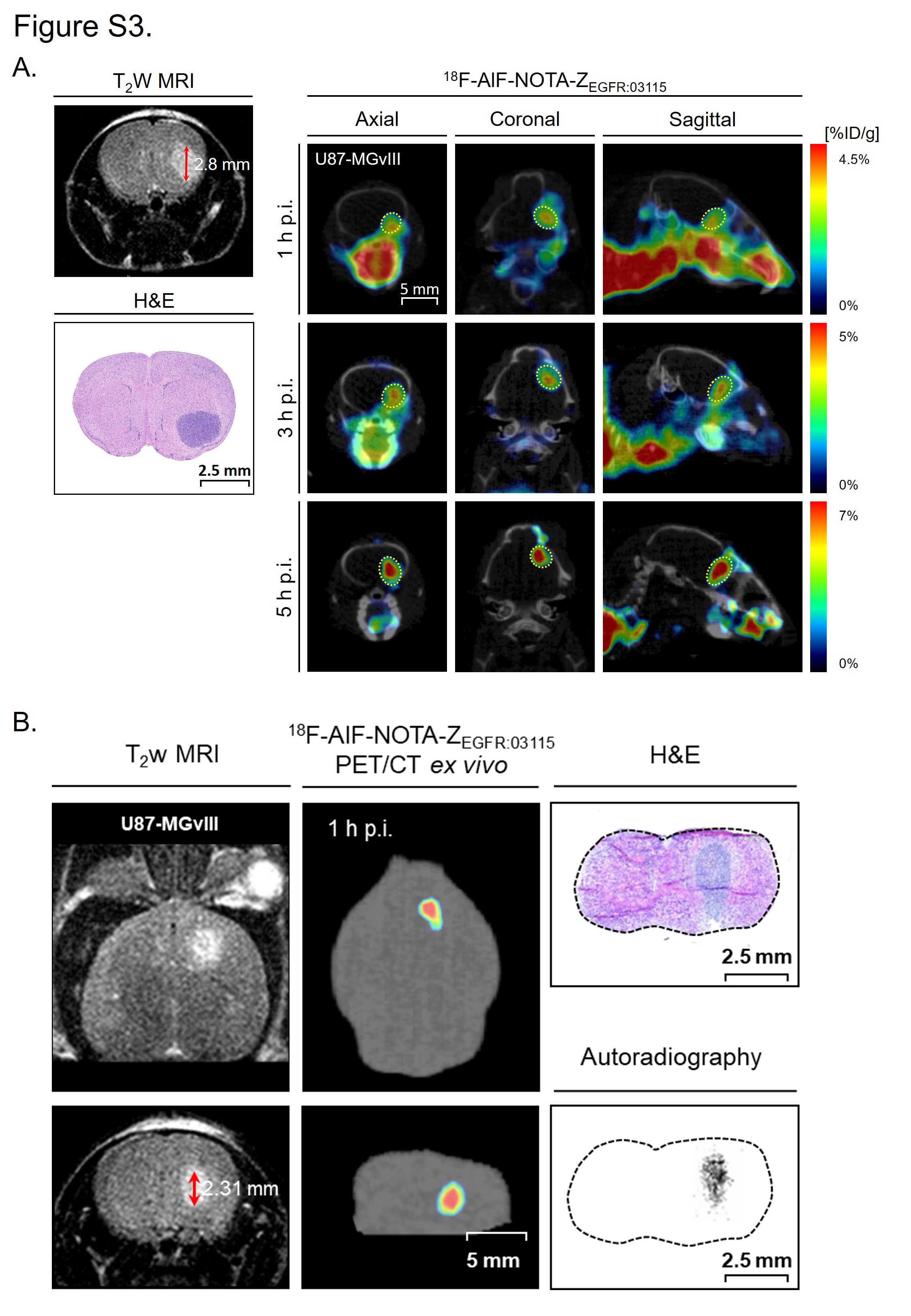
**

**Figure S3. Ability of ^18^F-AlF-NOTA-Z_EGFR:03115_ to accumulate in orthotopic U87-MGvIII tumours. (A**) *In vivo* uptake of the ^18^F-AlF-NOTA-Z_EGFR:03115_ in the U87-MGvIII orthotopic model. *In vivo* axial T_2_-weighted MRI image and corresponding Haematoxylin/Eosin staining of the orthotopic U87-MGvIII tumour 7 days after cells engraftment. Axial, coronal and sagittal *in vivo* PET/CT images of a representative mouse 1, 3 and 5 hours post-iv injection of ~2.4 MBq of the radiotracer. **(B) ^1^**^8^F-AlF-NOTA-Z_EGFR:03115_ uptake. *In vivo* T_2_-weighted MRI image on the coronal and axial plan (tumour diameter 2.31 mm) with corresponding *ex vivo* PET/CT acquisitions showing accumulation of ^18^F-AlF-NOTA-Z_EGFR:03115_ (5 μg co-injected with 7 μg of Z_EGFR:03115_; 2.43±0.15 MBq/mouse) one hour post-injection. Comparison of the Haematoxylin/Eosin staining and the autoradiography of corresponding frozen section.

**
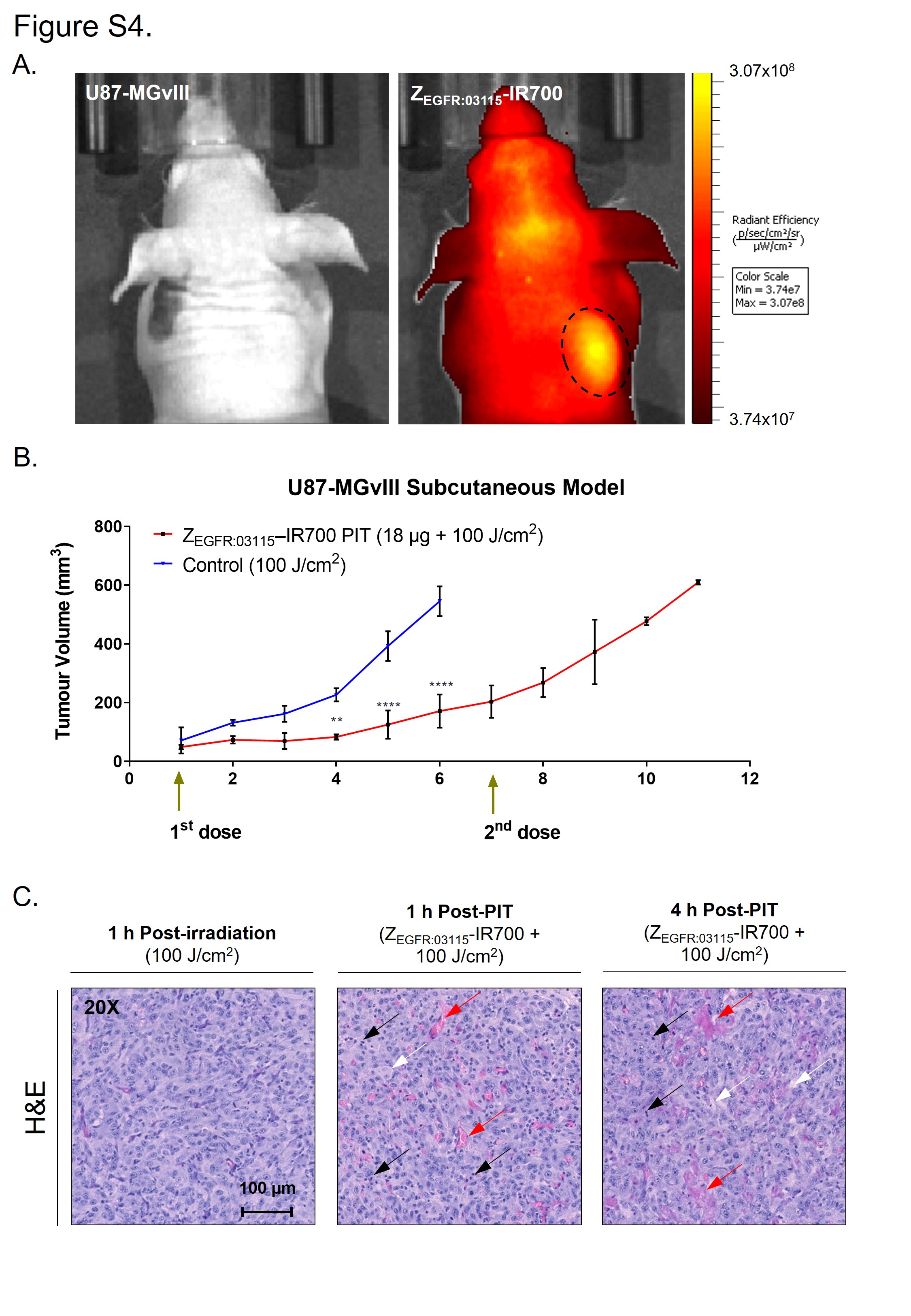
**

**Figure S4. *In vivo* EGFR-targeted PIT in U87-MGvIII subcutaneous tumours.** PIT efficacy study on a U87-MGvIII subcutaneous model. **(A)** Near-infrared imaging of a representative mouse bearing a subcutaneous U87-MGvIII tumour, 1 h after an i.v. injection of 18 μg of Z_EGFR:03115_-IR700. **(B)** Tumour volume measurements from day 1 to 11 after treatment initiation (n=3 per group, data presented as mean ± SD). Control mice were irradiated with 100 J/cm^2^. PIT treated mice received an i.v. injection of 18 μg of Z_EGFR:03115_-IR700 + 100 J/cm^2^ on day 1 and day 7 **(C)** Enlarged Haematoxylin/Eosin staining accurately indicating damage-related areas (20x). Red arrows indicating haemorrhagic areas, black arrows indicating pyknosis and apoptotic bodies and white arrows indicating necrotic areas.

**
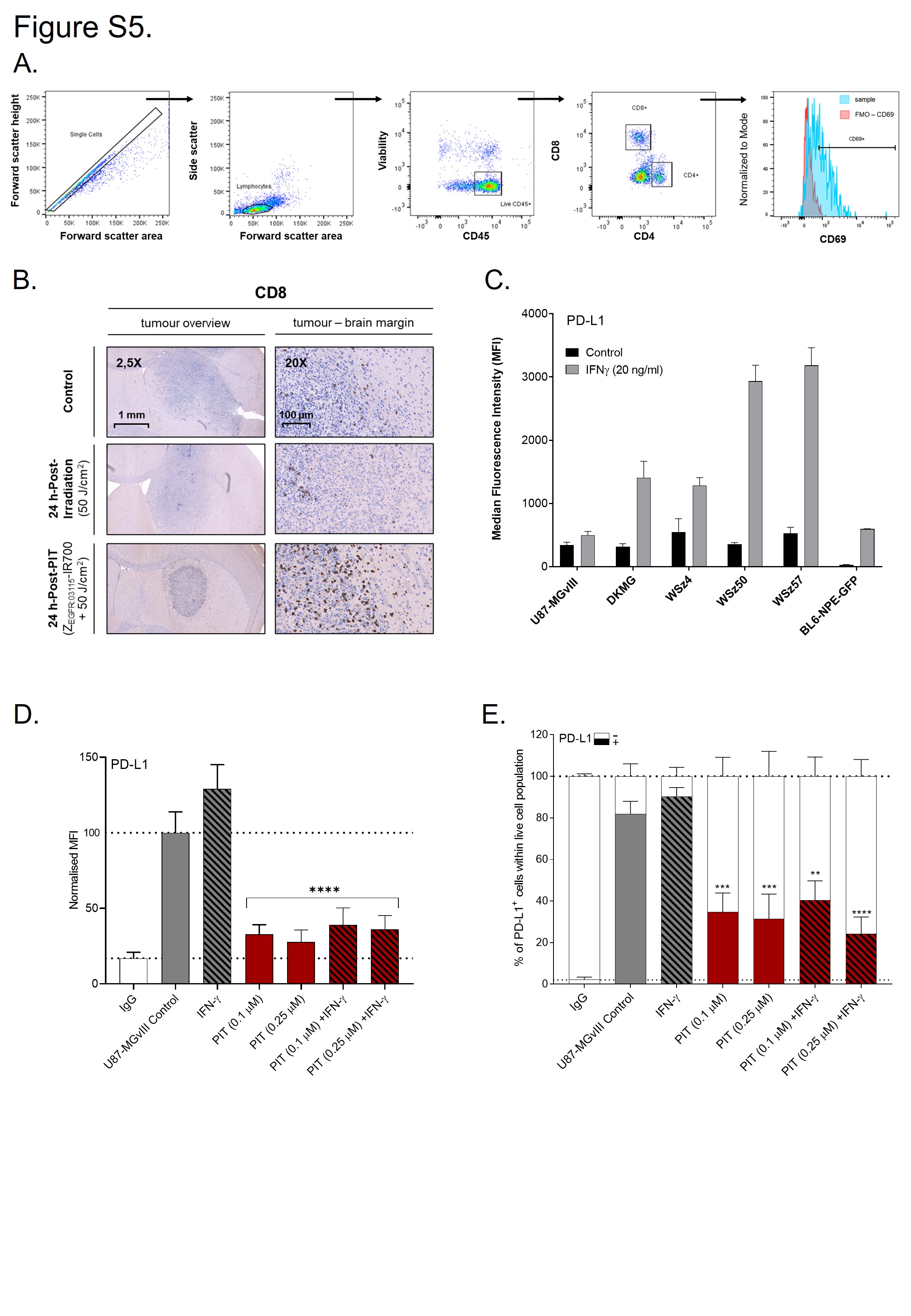
**

**Figure S5. Immune response to PIT. (A)** Gating strategy used to determine CD4, CD8 and CD69 positivity in T-cells (gated on CD45+). **(B)** Representative immunostaining of CD8+ the BL6-NPE-GFP brain tumours (2,5x & 20x) 24 h post-treatment. Treated mice were injected i.v. with 18 μg of the Z_EGFR:03115_-IR700 conjugate and irradiated with 50 J/cm^2^. **(C)** PD-L1 expression levels detected by flow cytometry in a panel of GBM cell lines with and without IFNγ (20 ng/ml) stimulation. **(D-E)** PD-L1 expression level on the surface of cell membrane and **(D)** percentage of PD-L1-positive cells (*coloured bars*) within live populations of U87-MGvIII cells as measured 4 h post-treatment by flow cytometry. PIT conditions: 1 h incubation with Z_EGFR:03115_-IR700 (0.1-0.25 μM) with or without 24 h pre-incubation IFNγ (20 ng/mL) and 16 J/cm^2^ light dose. The results were normalised to the control cells and presented as mean ± SEM (n=5). Statistical significance in comparison to the control (untreated) group was determined using ANOVA with Dunnett’s *post hoc* test. ****p≤0.0001, ***p≤0.001, **p≤0.01.
